# Building psychological resources for green organizational citizenship behavior: a broaden-and-build perspective on empowerment, environmental passion, and leaders’ emotional intelligence

**DOI:** 10.3389/fpsyg.2026.1868718

**Published:** 2026-08-18

**Authors:** Liqing Zhong, Juhee Hahn

**Affiliations:** 1 The Graduate School, Chung-Ang University, Seoul, Republic of Korea; 2 Department of Business Management, Chung-Ang University, Seoul, Republic of Korea

**Keywords:** broaden-and-build theory, Chinese new energy enterprises, empowerment, environmental passion, green organizational citizenship behavior, green transformational leadership, leaders’ emotional intelligence, multilevel analysis

## Abstract

Green organizational citizenship behavior is essential for translating corporate sustainability strategies into everyday work practices. Although green transformational leadership has been widely examined as an antecedent of employee green behavior, less is known about why it encourages employees to go beyond formal job duties and when this effect becomes stronger. Drawing on Broaden-and-Build Theory, this study develops a psychological resource model linking team-level green transformational leadership to green organizational citizenship behavior through employees’ cognitive-motivational and affective resources. Specifically, we examine general psychological empowerment (hereafter, empowerment) and environmental passion as complementary psychological resources, and leaders’ emotional intelligence (LEI), assessed through employee ratings, as a socio-emotional boundary condition. Multilevel path analysis was conducted using matched multi-source, three-wave time-lagged survey data from 517 employees nested within 77 teams in 10 Chinese new energy enterprises. The results showed that green transformational leadership was positively related to green organizational citizenship behavior. Empowerment and environmental passion mediated this relationship, while LEI strengthened both the green transformational leadership–green organizational citizenship behavior relationship and the empowerment–green organizational citizenship behavior relationship. These findings extend green leadership research by showing that a domain-specific leadership behavior can promote discretionary green behavior through both a general work-related cognitive-motivational resource and a domain-specific affective resource, and that this process becomes stronger when leaders are perceived as emotionally intelligent.

## 1 Introduction

Organizations increasingly rely on employees to support environmental goals in their daily work. Green organizational citizenship behavior (GOCB) refers to voluntary environmental behavior that goes beyond formal job requirements and is not directly required or rewarded by the organization (Boiral, 2009; Boiral and Paillé, 2012). Such behavior is important because formal environmental management systems alone cannot ensure that sustainability strategies are enacted in everyday work. Employees also need to take initiative, support green practices, and persist in environmentally responsible actions beyond their assigned duties. However, less is known about what psychological resources enable employees to willingly go beyond formal job requirements and sustain such green citizenship behavior.

Leadership is a central antecedent of employee green behavior. Green transformational leadership (GTL) is especially relevant because it emphasizes the articulation of a green vision, the modeling of pro-environmental values, and the encouragement of followers to pursue environmental goals beyond minimum expectations (Chen and Chang, 2013; Robertson and Barling, 2013; Robertson, 2018). Prior studies have shown that GTL can promote employee green behavior, green creativity, and other sustainability-related outcomes (Abourokbah et al., 2024; Zaid and Yaqub, 2024). Nevertheless, the theoretical explanation of how GTL promotes discretionary green behavior remains fragmented. Existing studies differ in their focal outcomes, mediating mechanisms, and boundary conditions, suggesting the need for a more coherent theoretical explanation of why, how, and when GTL leads to GOCB (Zacher et al., 2024).

The present study draws on Broaden-and-Build Theory to organize the proposed relationships among GTL, psychological resources, and GOCB. The theory was originally developed to explain how positive emotions broaden individuals’ momentary thought–action repertoires and, over time, contribute to the development of enduring personal resources (Fredrickson, 2001; Fredrickson et al., 2008). In organizational settings, supportive and meaningful work experiences may similarly create conditions that are conducive to psychological resource development (Ilies et al., 2024). In the context of green leadership, GTL can be understood as such a positive leadership context because it communicates a meaningful environmental vision, models environmentally responsible conduct, and encourages employee initiative. These leadership behaviors may shape how employees appraise environmental contribution and may support the development of psychological resources relevant to discretionary green action. In the present study, the broadening of employees’ environmental orientation constitutes the theoretical logic underlying these relationships rather than a separately measured mechanism.

Prior GTL research has identified a range of cognitive, motivational, and affective mechanisms linking green leadership with employee green behavior, including environmental attitude, green dedication, green intrinsic motivation, green psychological empowerment, and environmental passion (Li et al., 2020; Khan and Khan, 2022; Kaya and Atsan, 2025; Rietze et al., 2025; Cao et al., 2026). These studies provide important insights into specific pathways through which green leadership may influence employee behavior. Broaden-and-Build Theory offers a useful organizing perspective for examining these mechanisms as part of a broader psychological resource-development process. Building on this perspective, the present study distinguishes empowerment as a domain-general cognitive-motivational resource from environmental passion as a domain-specific affective resource. This distinction allows the study to examine how broad work-related agency and environmental affect may jointly support employees’ discretionary green behavior.

First, although prior research has examined the relationship between GTL and broad forms of employee green behavior, GOCB deserves more specific theoretical attention. GOCB is not simply another label for pro-environmental behavior. It is discretionary, extra-role, and sustained beyond formal task requirements. Therefore, it requires a stronger psychological explanation than behaviors that are already included in formal job roles or organizational procedures. Employees must see environmental action as meaningful, feel capable of making a difference, and remain willing to invest additional effort. Although some studies have examined GTL in relation to organizational citizenship behavior for the environment or related forms of environmental citizenship behavior (Khan and Khan, 2022; Li et al., 2023; Liu and Yu, 2023), less is known about how team-level GTL shapes employees’ GOCB through specific psychological resource-building processes.

Second, existing research has often explained the effects of GTL from either a cognitive-motivational perspective or an affective perspective, but has rarely examined how these two types of psychological resources jointly support discretionary green behavior. In the present study, empowerment represents a general cognitive-motivational state in which employees experience meaning, competence, self-determination, and impact in their work (Seibert et al., 2011). As a broad work-related resource, empowerment provides employees with the agency and confidence needed to initiate action beyond formal role requirements. When GTL makes environmental contributions salient, valued, and legitimate, employees may direct this general sense of agency toward GOCB. Environmental passion, by contrast, captures employees’ positive affective attachment to environmental activities and explains why they are emotionally energized to sustain green efforts beyond formal job requirements. From a broaden-and-build perspective, GTL may therefore foster two complementary psychological resources: empowerment as a general cognitive-motivational resource and environmental passion as a domain-specific affective resource. Examining these resources together enables the study to explain how broad work-related agency and environmental affect jointly support GOCB.

Third, the relationships of GTL and empowerment with GOCB may depend on how green leadership messages are communicated and received. GTL reflects the environmental content of leadership, including the vision, values, and behavioral expectations conveyed by leaders. However, how employees interpret and respond to these messages may also depend on the socio-emotional quality of leader–follower interactions. Leaders’ emotional intelligence (LEI) may play an important role in this process. Prior research indicates that LEI is associated with trust, interaction quality, and favorable employee outcomes (Ayoko et al., 2023). In green management contexts, leaders’ emotional intelligence has also been associated with employee green behavior and GOCB (Hu et al., 2023). In the present study, LEI is assessed through employee ratings and therefore reflects employees’ perceptions of their leader’s general emotional intelligence rather than an objective ability-based EI score. Such perceptions may influence whether employees experience green leadership messages as sincere, credible, and supportive. LEI may therefore provide a favorable socio-emotional context in which green values and behavioral expectations are more effectively communicated, understood, and accepted, thereby strengthening the GTL–GOCB and empowerment–GOCB relationships.

Based on these arguments, this study develops and tests a multilevel psychological resource-building model of GTL and GOCB. Specifically, we propose that team-level GTL promotes employees’ GOCB by fostering complementary cognitive-motivational and affective resources. Empowerment is examined as a domain-general work-related resource, whereas environmental passion is examined as a domain-specific affective resource. LEI is examined as a socio-emotional boundary condition. Following recent arguments on theory development, a stronger theoretical contribution should not merely add variables or combine multiple theoretical perspectives, but should clarify the relationships proposed by a focal theory, the intervening mechanisms that may connect antecedents and outcomes, and the conditions under which those relationships are expected to hold (Wagner and Berger, 1985; Cronin et al., 2021). This study makes three contributions. First, it applies Broaden-and-Build Theory to the green leadership context by conceptualizing GTL as a positive leadership context that may encourage employees to view environmental contribution as a meaningful domain for discretionary action and may create conditions conducive to psychological resource development. Second, it distinguishes two complementary resources with different levels of domain specificity: empowerment provides broad work-related agency, whereas environmental passion provides affective attachment to environmental activities. Third, it identifies LEI as a proximal socio-emotional condition under which the relationships of GTL and empowerment with GOCB may become stronger. Together, these contributions clarify how domain-specific leadership, domain-general work-related agency, domain-specific environmental affect, and the socio-emotional leadership context are jointly associated with discretionary green behavior.

The empirical context of this study is Chinese new energy enterprises. This context is appropriate because environmental goals are highly salient in new energy firms, and employees’ voluntary green contributions may directly affect whether organizational sustainability strategies are implemented in daily work. In this setting, formal environmental goals need to be translated into daily discretionary behavior through team-level leadership and employee psychological resources. The study uses matched multi-source, three-wave time-lagged data from 517 employees nested within 77 teams in 10 Chinese new energy enterprises. On this basis, the study addresses the following research questions:

RQ1: How does team-level GTL influence employee GOCB?

RQ2: Do empowerment and environmental passion explain how GTL is translated into employee GOCB?

RQ3: Does LEI strengthen the effects of GTL and empowerment on GOCB?

## 2 Theoretical background and hypotheses

### 2.1 Theoretical framework: a broaden-and-build perspective

Drawing on Broaden-and-Build Theory, this study adopts a resource-development perspective to organize the proposed relationships among GTL, empowerment, environmental passion, LEI, and GOCB. Broaden-and-Build Theory was originally developed to explain how positive emotions broaden individuals’ momentary thought–action repertoires and, over time, contribute to the development of enduring personal resources (Fredrickson, 2001; Fredrickson et al., 2008). The present application focuses on the theory’s resource-building logic by examining how a positive leadership context may be associated with psychological resources that support discretionary green behavior. Within this framework, the broadening of employees’ environmental orientation is treated as a proposed theoretical process rather than as a separately operationalized construct.

GTL is conceptualized as a positive leadership context rather than as a positive emotion or a personal resource. By communicating an inspiring environmental vision, modeling environmentally responsible conduct, and encouraging employee initiative, GTL may create supportive work experiences that are conducive to psychological resource development (Ilies et al., 2024). The resource component of the model is represented by empowerment and environmental passion. Empowerment constitutes a domain-general cognitive-motivational resource reflected in employees’ experiences of meaning, competence, self-determination, and impact at work (Spreitzer, 1995; Seibert et al., 2011). Environmental passion constitutes a domain-specific affective resource reflected in employees’ positive attachment to and enthusiasm for environmental activities (Robertson and Barling, 2013; Li et al., 2020; Cao et al., 2026). GOCB is examined as the focal behavioral outcome, whereas LEI is conceptualized as a socio-emotional boundary condition.

Accordingly, the proposed framework links a domain-specific leadership context with two complementary psychological resources that differ in their level of domain specificity. Empowerment provides employees with a broad sense of agency in their work, whereas environmental passion provides affective attachment to environmental activities. LEI further captures the socio-emotional context that may shape whether GTL and empowerment are more strongly associated with GOCB. This framework therefore provides the theoretical basis for the relationships developed in the following hypotheses, while treating the broadening of employees’ environmental orientation as an explanatory proposition rather than a directly measured mechanism.

### 2.2 GTL and GOCB

GOCB refers to voluntary environmental actions through which employees contribute to organizational environmental sustainability beyond formal job requirements (Boiral, 2009; Boiral and Paillé, 2012). Because such behavior is neither formally prescribed nor directly rewarded, employees’ engagement in GOCB may depend partly on whether environmental contributions are perceived as meaningful, valued, and supported in the work context. Explaining GOCB therefore requires attention to leadership conditions that make voluntary environmental action salient and legitimate.

GTL represents a particularly relevant leadership condition. As an environmentally specific form of transformational leadership, GTL involves articulating an inspiring environmental vision, modeling environmentally responsible conduct, and encouraging employees to pursue environmental objectives beyond minimum expectations (Chen and Chang, 2013; Robertson and Barling, 2013; Robertson, 2018). Through these behaviors, green transformational leaders emphasize the importance of environmental responsibility and signal that voluntary green contributions are valued within the team.

According to Broaden-and-Build Theory, positive experiences can widen individuals’ attentional scope and thought–action repertoires, enabling them to notice and consider possibilities beyond habitual responses (Fredrickson, 2001; Fredrickson and Branigan, 2005). In the green leadership context, GTL may create supportive and meaningful work experiences by framing environmental contribution as valuable and legitimizing employee initiative. Such experiences may direct employees’ attention beyond prescribed tasks toward opportunities for environmental improvement and make a broader repertoire of discretionary green responses cognitively available.

Moreover, recent empirical research provides convergent support for this expectation. GTL has been associated with greater green mindfulness and environmental awareness, indicating that environmentally focused leadership may heighten employees’ attention to environmental cues and opportunities for action (Fu et al., 2025; Oktaysoy et al., 2025). Other studies have linked GTL with employee green behavior, pro-environmental behavior, and organizational citizenship behavior for the environment (Liu and Yu, 2023; Li et al., 2023; Zaid and Yaqub, 2024; Kaya and Atsan, 2025; Cao et al., 2026). Therefore, we propose the following hypothesis:

*Hypothesis 1: GTL is positively related to GOCB.*

### 2.3 GTL and empowerment

Empowerment refers to a general cognitive-motivational state in which employees experience meaning, competence, self-determination, and impact at work (Spreitzer, 1995). Meaning reflects the fit between employees’ work roles and their personal values; competence refers to confidence in their ability to perform work activities successfully; self-determination reflects discretion in initiating and regulating work behavior; and impact refers to the perceived ability to influence work outcomes (Hu, 2025). Empowerment therefore represents more than the formal delegation of authority. It captures employees’ broader psychological experience of agency in their work. Meta-analytic evidence indicates that empowerment is shaped by contextual factors, including leadership and work design, and is positively associated with employee attitudes, performance, and proactive behavior (Seibert et al., 2011).

The resource-building logic of Broaden-and-Build Theory provides a basis for explaining how such work-role appraisals may develop. Psychological resources may emerge from work experiences that enhance the perceived significance of employees’ roles, reinforce their capabilities, and expand their perceived possibilities for action. GTL may provide such experiences by embedding employees’ everyday work within a valued environmental purpose and positioning them as capable contributors to collective environmental objectives. An inspiring environmental vision may strengthen the meaning attached to employees’ broader work roles, while intellectual stimulation and encouragement of initiative may reinforce competence and self-determination. Emphasizing the significance of employees’ contributions may also make their influence on collective outcomes more visible, thereby strengthening perceived impact (Chen and Chang, 2013; Robertson and Barling, 2013).

Evidence from recent empowerment research supports this expectation. Meta-analytic findings identify leadership as an important contextual antecedent of psychological empowerment and show that transformational leadership is positively associated with employees’ empowerment perceptions (Schermuly et al., 2022; Llorente-Alonso et al., 2024). In the environmental leadership context, Yang et al. (2025) similarly found a positive association between environmentally specific transformational leadership and general psychological empowerment. Unlike green psychological empowerment, which anchors meaning, competence, self-determination, and impact specifically to environmental work, empowerment in the present study concerns employees’ broader experience of agency across their work roles (Rietze et al., 2025). Therefore, we propose the following hypothesis:

*Hypothesis 2: GTL is positively related to empowerment*.

### 2.4 Mediating role of empowerment between GTL and GOCB

Whether employees act on opportunities for environmental improvement may depend partly on whether they believe they have sufficient discretion and influence to make a meaningful difference. Empowerment may support such judgments by strengthening employees’ perceptions that they can shape how their work is performed and influence relevant work outcomes. These perceptions may be particularly pertinent to GOCB, which includes voluntarily initiating environmental improvements, helping colleagues adopt more environmentally responsible practices, and participating in organizational environmental activities beyond formal requirements (Boiral and Paillé, 2012). Consistent with this reasoning, recent meta-analytic evidence indicates a positive association between psychological empowerment and organizational citizenship behavior (Llorente-Alonso et al., 2024).

From a broaden-and-build perspective, a supportive green leadership context may widen employees’ perceived possibilities for action by making environmental improvement a more salient and legitimate avenue for exercising work-related agency (Ilies et al., 2024). Empowerment, in turn, may provide the general sense of meaning, competence, self-determination, and impact needed to pursue these possibilities. In this way, GTL provides domain-specific environmental direction, whereas empowerment represents domain-general work-related agency, offering a theoretical basis for an indirect positive relationship between GTL and GOCB.

A closely related pattern has also emerged in recent green leadership research. Yang et al. (2025) found that general psychological empowerment partly accounted for the positive association between environmentally specific transformational leadership and green voice behavior. Although green voice and GOCB differ in their specific behavioral expression, both involve discretionary environmental contributions beyond prescribed job duties. Against this background, we propose the following hypothesis:

*Hypothesis 3: Empowerment mediates the relationship between GTL and GOCB.*

### 2.5 GTL and environmental passion

Passion arises when individuals regard an activity as personally important and are willing to invest sustained time and energy in it (Vallerand et al., 2003). Applied to the environmental domain, environmental passion captures employees’ positive and enduring affective attachment to environmental activities at work (Robertson and Barling, 2013). It represents more than a temporary emotional reaction to a particular environmental event. Employees with stronger environmental passion are likely to experience environmental involvement as personally significant, emotionally engaging, and worthy of continued commitment.

Green transformational leaders may shape how employees emotionally appraise environmental activities. By articulating an inspiring environmental vision, demonstrating personal commitment to sustainability, recognizing employees’ green initiatives, and connecting individual contributions with collective environmental goals, such leaders may make environmental involvement more personally consequential and emotionally rewarding. The resource-building logic of Broaden-and-Build Theory suggests that recurring positive and meaningful experiences can contribute to the development of more enduring psychological resources (Fredrickson, 2001; Fredrickson et al., 2008). In this context, repeated experiences of purpose, encouragement, and accomplishment surrounding environmental activities may deepen employees’ affective attachment to those activities. GTL may therefore be positively associated with environmental passion by creating a leadership context in which environmental engagement is experienced as a valued and meaningful part of work rather than merely as compliance with organizational expectations.

Recent green leadership research provides further support for this expectation. Environmentally specific transformational leadership has been positively associated with harmonious environmental passion (Iqbal et al., 2023). Similar positive relationships have been reported between GTL and employee green passion, as well as between team-level GTL and employees’ environmental passion in cross-level settings (Abourokbah et al., 2024; Cao et al., 2026). Although these studies use somewhat different labels for environmentally oriented passion, their findings consistently associate environmentally focused transformational leadership with stronger affective investment in environmental activities. Therefore, we propose the following hypothesis:

*Hypothesis 4: GTL is positively related to environmental passion.*

### 2.6 Mediating role of environmental passion

Employees’ continued involvement in voluntary environmental activities may depend partly on the personal and emotional significance they attach to those activities. Environmental passion may make environmental engagement intrinsically meaningful and emotionally rewarding, thereby increasing employees’ willingness to devote sustained attention and effort to green initiatives beyond their prescribed responsibilities (Iftikhar et al., 2024). As a relatively enduring affective attachment, environmental passion may be particularly relevant when environmental contributions require persistence but offer few immediate formal rewards. Recent evidence has positively associated environmental passion with organizational citizenship behavior for the environment and related forms of voluntary environmental behavior (Alwheshi et al., 2024; Iftikhar et al., 2024).

This affective connection is consistent with the resource-building logic of Broaden-and-Build Theory. GTL may imbue environmental contribution with purpose and positive significance, while environmental passion may sustain this significance as a domain-specific affective resource. Employees who develop a stronger emotional attachment to environmental activities may be more inclined to maintain their involvement when green action demands additional time, persistence, or personal initiative. Environmental passion may therefore connect an environmentally meaningful leadership context with GOCB by sustaining employees’ affective investment in voluntary environmental action.

A similar indirect pattern has been documented in green leadership research. Environmental passion has accounted for an indirect relationship between environmentally specific transformational leadership and employees’ pro-environmental behavior (Li et al., 2020). Harmonious environmental passion has likewise been identified as an intervening variable between environmentally specific transformational leadership and organizational citizenship behavior for the environment (Iqbal et al., 2023). More recent cross-level evidence also indicates an indirect relationship between team-level GTL and employee green behavior through environmental passion (Cao et al., 2026). Although these studies differ in their precise passion and behavioral measures, their findings support the expectation that environmental passion may provide an affective pathway connecting GTL with discretionary environmental action. Accordingly, we propose the following hypothesis:

*Hypothesis 5: Environmental passion mediates the relationship between GTL and GOCB.*

### 2.7 Moderating role of LEI between GTL and GOCB

In the present study, LEI refers to employees’ shared perceptions of their immediate leader’s general emotional intelligence, including the perceived abilities to understand the leader’s own emotions and those of others, use emotions to facilitate goal pursuit, and regulate emotions in workplace interactions (Wong and Law, 2002; Mayer et al., 2016). Because LEI is assessed through employee ratings, it captures how employees experience their leader’s emotional competence rather than an objective ability-based EI score. This perceptual framing is theoretically relevant because employees’ responses to leadership may depend not only on the content of leadership messages but also on the interpersonal manner in which those messages are conveyed.

GTL communicates an environmental vision, environmental values, and behavioral expectations and encourages employees to contribute to organizational sustainability beyond formal role requirements. In the present framework, LEI represents a socio-emotional boundary condition that may shape how these messages are communicated, interpreted, and accepted. Although green organizational climate, green HRM practices, and team environmental norms may also influence employee green behavior, these factors represent relatively distal structural or normative conditions (Zacher et al., 2024; Dwumah et al., 2025; Zafar et al., 2025). LEI is more proximal to the leader–follower interactions through which employees experience GTL. Leaders who are perceived as emotionally intelligent may be better able to recognize employees’ concerns, adapt their communication to employees’ emotional responses, and present environmental expectations in a supportive rather than pressuring manner (Miao et al., 2018; Ayoko et al., 2023). Consequently, employees may regard GTL messages as more authentic and credible and may be more inclined to engage in GOCB.

Conversely, when leaders are perceived as lower in emotional intelligence, they may still communicate environmental objectives but may be less effective in responding to employees’ uncertainty, emotional reactions, or concerns about additional green effort. Similar environmental expectations may therefore be experienced as less supportive or more demanding, reducing the strength of the positive association between GTL and GOCB. This argument is consistent with meta-analytic evidence associating leader emotional intelligence with subordinates’ organizational citizenship behavior (Miao et al., 2018), research indicating that leaders’ emotional intelligence conditions the relationship between leadership style and citizenship behavior (Abdullahi et al., 2020), and green management research linking perceived leader emotional intelligence with employee green behavior and GOCB (Hu et al., 2023). Based on this reasoning, we propose the following hypothesis:

*Hypothesis 6: LEI positively moderates the relationship between GTL and GOCB, such that the relationship is stronger when LEI is high.*

### 2.8 Moderating role of LEI between empowerment and GOCB

Feeling capable of influencing one’s work does not eliminate the interpersonal uncertainty associated with discretionary action. Empowered employees may possess the discretion and confidence needed to initiate change, yet engaging in GOCB can require them to propose environmental improvements, mobilize colleagues’ cooperation, or devote effort to initiatives that fall outside their formally assigned responsibilities. Whether employees express their work-related agency through such behavior may therefore depend on how they expect their leader to interpret and respond to initiative. When constructive action is likely to be recognized rather than misconstrued as interference or inappropriate role expansion, empowerment may be more strongly associated with GOCB (Llorente-Alonso et al., 2024; Xu et al., 2024).

This relational uncertainty makes LEI particularly relevant. Leaders who are perceived as emotionally intelligent may be better able to recognize the constructive intentions underlying employee initiatives, regulate their own reactions, and respond to new ideas in a considerate and constructive manner. Multilevel evidence has associated leader emotional intelligence with trust in supervisors (Lee et al., 2023), while recent research has highlighted communication, transparency, and trust as important features of emotionally intelligent leadership (Ertiö et al., 2024). Leadership emotional intelligence has also been linked to psychological safety and team innovation in a multilevel setting (Xing et al., 2026). In teams characterized by higher LEI, empowered employees may therefore be more confident that their voluntary environmental initiatives will be understood, taken seriously, and evaluated according to their constructive intent.

Within the broaden-and-build framework adopted in this study, empowerment represents a cognitive-motivational resource, whereas LEI specifies a socio-emotional condition under which the relationship between this resource and discretionary green action may become stronger. When leaders are perceived as lower in emotional intelligence, employees may retain a sense of agency but experience greater ambiguity regarding the interpersonal consequences of acting beyond prescribed duties. More favorable shared perceptions of LEI may reduce this ambiguity and make employees more willing to apply their discretion and perceived influence to environmental initiatives. Accordingly, the following hypothesis is proposed:

*Hypothesis 7: LEI positively moderates the relationship between empowerment and GOCB, such that the relationship is stronger when LEI is high.*

**Figure 1:**
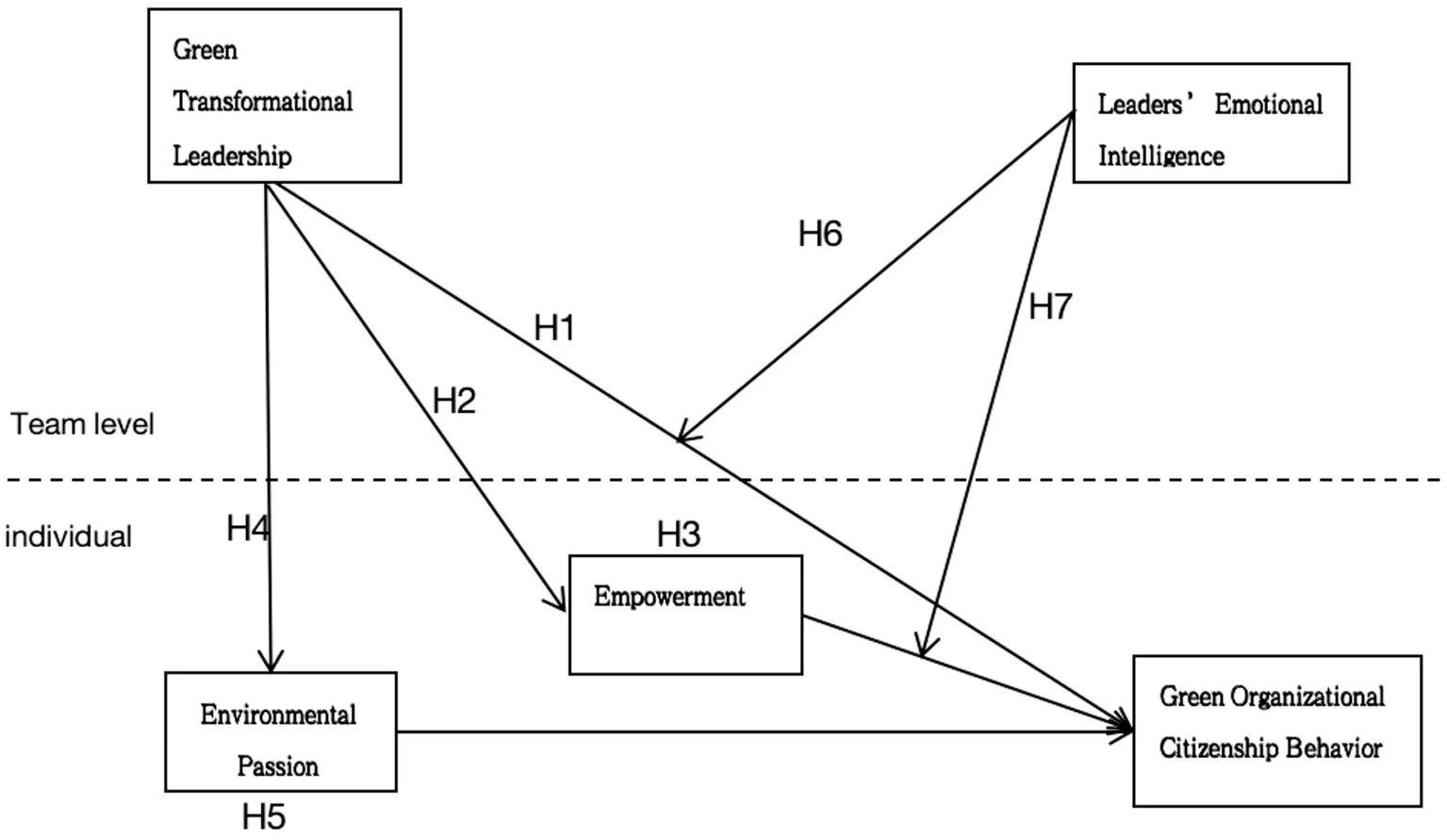
Conceptual model of the hypothesized multilevel relationships.

Figure 1 depicts our research model, which shows how the hypotheses relate at the team and individual levels.

## 3 Method

### 3.1 Sample and procedure

We conducted a three-wave, multi-source, time-lagged survey between September 15 and November 4, 2023, among new energy enterprises located in Guangdong, Jiangsu, and Zhejiang, China. The participating enterprises operated in several areas of the new energy sector, including energy generation, electric vehicles and charging infrastructure, new energy technology research and development, energy management and intelligent systems, and environmental project development. This empirical setting was appropriate because environmental objectives are particularly salient in the new energy sector, while many environmentally beneficial employee behaviors remain discretionary and are enacted within supervisor-led work teams.

We employed a purposive, non-probability organizational sampling strategy, followed by intact-team sampling within the participating enterprises. Potential organizations were identified through provincial industry association rosters and publicly available industry databases. To be eligible, an organization was required to operate in the new energy sector, maintain stable team-based work structures with identifiable formal team leaders, and be able to participate in all three waves of matched data collection. Organizations were excluded if they did not operate in the focal sector, lacked stable team structures or formal immediate supervisors, or were unable to support the three-wave matched design.

We initially contacted the human resources departments of 32 organizations. Eight organizations explicitly declined participation, 14 did not respond, and 10 granted the research team access, yielding an organizational participation rate of 31.25%. In each participating enterprise, an authorized HR representative served as the organizational liaison, provided rosters of eligible intact teams, and assisted with communication and questionnaire distribution. Team leaders were formal immediate supervisors responsible for assigning work and evaluating employee performance. Employees were eligible to participate if they worked full-time in the focal team and had at least 6months of organizational tenure, increasing the likelihood that they had sufficient exposure to the same leader and team context.

Before the formal survey, we conducted three preliminary group discussions with 20 team leaders from the participating enterprises to pretest the questionnaire. The sessions were used solely to evaluate item clarity, contextual appropriateness, and the feasibility of the survey procedures rather than to generate qualitative data. Accordingly, the discussions were not audio-recorded, transcribed, or formally coded. Based on participants’ feedback, only minor wording and procedural adjustments were made. None of the participants involved in the pretesting sessions was included in the formal study sample.

The survey was administered in Chinese. Following a standard translation and back-translation procedure, the original English-language scales were translated into Chinese and independently back-translated into English by two bilingual researchers. The translated and back-translated versions were compared to identify and resolve any discrepancies and to ensure semantic consistency before the questionnaire was finalized.

Participation in the formal survey was voluntary. Before accessing their respective questionnaires, participants viewed an electronic informed-consent statement describing the purpose of the study, the estimated completion time, confidentiality protections, the use of the data only in aggregate form, and their right to discontinue participation at any time without penalty. Participants provided documented written informed consent electronically by selecting an agreement option before accessing the survey items. Separate online questionnaires and survey links were used for team leaders and employees. The study protocol, including the preliminary questionnaire-pretesting discussions and the formal three-wave survey, was reviewed and approved by the Chung-Ang University Institutional Review Board on September 4, 2023 (approval no. CAU-20230915), before formal data collection began on September 15, 2023. The study comprised three measurement waves separated by intervals of approximately 2 weeks. In Wave 1, conducted from September 15 to September 22, 2023, employees evaluated their immediate leader’s green transformational leadership (GTL) and reported their own empowerment. In Wave 2, conducted from October 7 to October 14, 2023, the same employee cohort evaluated their leader’s emotional intelligence (LEI) and reported their environmental passion. In Wave 3, conducted from October 29 to November 4, 2023, team leaders evaluated the green organizational citizenship behavior (GOCB) of each participating employee. Non-identifying team and employee codes were used to match employee responses across Waves 1 and 2 with the corresponding employee-specific leader ratings collected in Wave 3.

LEI was measured in Wave 2 to temporally separate the two employee-rated evaluations of the same leader, namely GTL and LEI. This separation was intended to reduce immediate response consistency and same-occasion covariance. It was not intended to imply that LEI developed after or was caused by GTL, nor did it establish temporal change or causal ordering. Both measures captured employees’ accumulated experiences with the same leader, and LEI was conceptualized as a relatively stable, employee-perceived socio-emotional leadership context over the short data-collection period.

A total of 630 respondent records entered the data-screening and matching process. After 36 records lacking one or more required survey components were excluded, the final analytic sample consisted of 517 employees nested within 77 teams and the 77 team leaders who supervised those teams. All retained employee records contained complete Wave 1 and Wave 2 responses and a successfully matched Wave 3 leader rating. No partially completed responses were retained, and no missing-data imputation was performed. Because separate wave-specific invitation and completion counts were not retained in the archived administrative records, wave-specific response and attrition rates could not be calculated. In addition, enterprise identifiers were not retained in the de-identified analytic dataset; therefore, the numbers of teams and employees contributed by each enterprise could not be reliably reconstructed.

Among the 77 team leaders, 53 (68.83%) were male and 24 (31.17%) were female. Ten leaders (12.99%) were aged 20–30 years, 15 (19.48%) were aged 31–40 years, 33 (42.86%) were aged 41–50 years, and 19 (24.68%) were over 50 years old. Regarding education, 25 leaders (32.47%) held a junior college degree or below, 46 (59.74%) held a bachelor’s degree, and six (7.79%) held a master’s degree or above. Five teams (6.49%) had 1–8 members, 67 teams (87.01%) had 9–16 members, and five teams (6.49%) had 17–24 members. Team size was retained in the de-identified dataset as three ordered categories rather than as an exact headcount. Because exact team headcounts were unavailable, team size was represented in the analyses using the three ordered category scores. The highly uneven distribution across the categories should therefore be considered when interpreting the team-size coefficient.

Among the 517 employees, 268 (51.84%) were male and 249 (48.16%) were female. A total of 230 employees (44.49%) were aged 20–30 years, 189 (36.56%) were aged 31–40 years, 81 (15.67%) were aged 41–50 years, and 17 (3.29%) were over 50 years old. Regarding education, 231 employees (44.68%) held a junior college degree or below, 267 (51.64%) held a bachelor’s degree, and 19 (3.68%) held a master’s degree or above.

### 3.2 Measures

All items were rated on a five-point Likert scale ranging from 1 = strongly disagree to 5 = strongly agree.

GTL was assessed using a 6-item scale developed by Chen and Chang (2013). A sample item is “My leader of the green innovation project inspires the project members with environmental plans.”

GOCB was evaluated using the 7-item scale developed by Boiral and Paillé (2012). A sample item is “My subordinate voluntarily conducts environmental actions and initiatives in daily work activities.”

Empowerment was assessed using an 11-item scale developed by Hancer and George (2003). A sample item is “I have freedom in determining how to conduct my job.”

Environmental passion was captured with the 10-item scale developed by Robertson and Barling (2013). A sample item is “I am passionate about the environment.”

LEI was measured with the 16-item scale employed by Hu et al. (2023). A sample item is “My leader has a good sense of why he has certain feelings most of the time.”

### 3.3 Analytical strategy

Because employees were nested within work teams, this study adopted a multilevel analytical strategy. The final analytic sample consisted of 517 employees nested within 77 teams. GTL and LEI were conceptualized as team-level constructs because they represented employees’ shared evaluations of the same immediate team leader, whereas empowerment, environmental passion, and GOCB were treated as individual-level constructs. Unconditional null models were first estimated in Mplus 8.3 to partition the variance in environmental passion, empowerment, and GOCB into within-team and between-team components and to determine whether multilevel analysis was warranted.

Before aggregating employee-rated GTL and LEI to the team level, we examined whether aggregation was empirically justified. Following established recommendations for multilevel research, within-group agreement and between-group reliability were evaluated using Rwg, ICC(1), and ICC(2) (James et al., 1984; Klein and Kozlowski, 2000; LeBreton and Senter, 2008). Rwg reflects the extent to which employees within the same team provide similar evaluations of the focal construct, ICC(1) represents the proportion of variance attributable to team membership, and ICC(2) evaluates the reliability of team mean scores. GTL demonstrated adequate agreement and reliability, with Rwg = 0.836, ICC(1) = 0.419, and ICC(2) = 0.829. LEI likewise demonstrated acceptable aggregation indices, with Rwg = 0.815, ICC(1) = 0.346, and ICC(2) = 0.780. These results supported the aggregation of employee-rated GTL and LEI to the team level.

The measurement model was evaluated using conventional single-level confirmatory factor analysis in Mplus 8.3 based on the 517 matched employee-level records. GTL, empowerment, LEI, and environmental passion were reported by employees, whereas the GOCB indicators consisted of employee-specific ratings provided by the corresponding team leaders. Each leader-rated GOCB record was matched to the focal employee’s responses using the non-identifying employee code. The GTL and LEI indicators were analyzed before aggregation. Accordingly, the factor loadings reported in Table 1 represent single-level estimates rather than separate within-team or between-team estimates. Team clustering was not explicitly adjusted in the CFA. A full item-level multilevel CFA was not estimated because the between-team sample consisted of 77 teams, which was limited relative to the number of indicators and parameters required to estimate the complete measurement model separately at both levels. The single-level CFA was therefore used as a preliminary assessment of the proposed factor structure rather than as a test of distinct within-team and between-team measurement structures.

**Table 1:** Reliability and convergent validity.

Variable	Level	Source	Items	Alpha	Factor loading	CR	AVE
GTL	Team	Employee-rated	6	0.917	0.791–0.826	0.917	0.649
LEI	Team	Employee-rated	16	0.920	0.692–0.739	0.813	0.521
Empowerment	Individual	Employee-rated	11	0.946	0.761–0.804	0.946	0.615
Environmental Passion	Individual	Employee-rated	10	0.959	0.797–0.867	0.959	0.700
GOCB	Individual	Leader-rated	7	0.905	0.732–0.776	0.905	0.577

GTL, green transformational leadership; GOCB, green organizational citizenship behavior; LEI, leaders’ emotional intelligence. GTL and LEI were conceptualized as team-level constructs and aggregated from employee ratings for the multilevel path analyses. The reliability and convergent validity statistics reported in this table were estimated from the 517 pre-aggregation employee-level records. For LEI, the factor-loading range refers to the standardized loadings of its four first-order dimensions on the second-order LEI construct.

The hypothesized measurement model comprised five substantive constructs: GTL, LEI, empowerment, environmental passion, and GOCB. Model fit was evaluated using the comparative fit index (CFI), Tucker–Lewis index (TLI), root mean square error of approximation (RMSEA), and standardized root mean square residual (SRMR). CFI and TLI values above 0.90 and RMSEA and SRMR values below 0.08 were treated as indicating acceptable model fit (Browne and Cudeck, 1992; Hu and Bentler, 1999). Reliability and convergent validity were assessed using Cronbach’s *α*, composite reliability (CR), average variance extracted (AVE), and standardized factor loadings. Discriminant validity was evaluated using the Fornell–Larcker criterion and by comparing the hypothesized five-factor model with theoretically plausible alternative measurement models (Fornell and Larcker, 1981).

The hypotheses were tested using observed-variable, two-level path analysis in Mplus 8.3. The models were estimated using maximum likelihood estimation. The TWOLEVEL specification was used to account for the nesting of employees within teams and to decompose the variation in empowerment, environmental passion, and GOCB into within-team and between-team components. GTL and LEI were modeled at the between-team level. Environmental passion, empowerment, and GOCB were represented at the within-team level and were also allowed to vary at the between-team level. The mediator–outcome relationships were specified under a fixed-slope formulation.

GTL and LEI were grand-mean centered before the GTL × LEI interaction term was created. For the cross-level interaction between empowerment and LEI, both empowerment and LEI were grand-mean centered, and the empowerment × LEI product term was calculated before model estimation. This centering strategy preserved both within-team and between-team variation in empowerment and was consistent with the specification used to test whether the overall empowerment–GOCB relationship varied across levels of team-level LEI (Enders and Tofighi, 2007). Significant interactions were further examined using simple-slope analyses at one standard deviation below and above the mean of LEI. The corresponding conditional slopes, slope differences, standard errors, and 95% confidence intervals are reported in Table 2.

**Table 2:** Moderating effects of LEI.

Path	Level of LEI	Estimate	SE	P	95% CI lower bound	95% CI upper bound
GTL → GOCB	Low LEI	0.049	0.128	0.704	−0.203	0.300
	High LEI	0.485	0.107	p < 0.001	0.274	0.696
	Difference	0.436	0.181	0.016	0.081	0.791
EMP → GOCB	Low LEI	0.159	0.102	0.119	−0.041	0.358
	High LEI	0.457	0.088	p < 0.001	0.285	0.630
	Difference	0.299	0.152	0.049	0.001	0.596

GTL, green transformational leadership; EMP, empowerment; LEI, leaders’ emotional intelligence; GOCB, green organizational citizenship behavior; CI, confidence interval.

Indirect relationships were evaluated using the product-of-coefficients approach. Specifically, the GTL → empowerment coefficient was multiplied by the empowerment → GOCB coefficient to estimate the indirect relationship through empowerment, whereas the GTL → environmental passion coefficient was multiplied by the environmental passion → GOCB coefficient to estimate the indirect relationship through environmental passion. Because the team-level predictor–mediator paths were combined with mediator–outcome coefficients from the fixed-slope multilevel specification, the reported estimates should be interpreted as model-based cross-level indirect associations rather than as purely within-team or purely between-team indirect effects. Standard errors and confidence intervals for the indirect estimates were calculated in Mplus using the delta method through the MODEL CONSTRAINT and CINTERVAL specifications (Preacher et al., 2010).

Potential deviations from normality were evaluated before hypothesis testing. The absolute skewness values of the focal variables ranged from 0.42 to 0.87, and the absolute kurtosis values ranged from 0.31 to 0.96, indicating no severe departures from normality. The two-level model specification accounted for the clustering of employees within teams. Only records containing complete Wave 1 and Wave 2 employee responses and a successfully matched Wave 3 leader rating were retained. No partially completed records were included, and no missing-data imputation was performed.

The control variables were included as robustness covariates rather than selected solely because of statistically significant zero-order correlations (Becker, 2005; Bernerth and Aguinis, 2016). At the individual level, employee gender, age, and education were considered because demographic characteristics may be associated with environmental attitudes, discretionary behavior, and employees’ opportunities to engage in voluntary workplace activities. At the team level, leader gender, age, and education were considered because leader characteristics may covary with leadership behavior and employees’ perceptions of leadership. Team size was included because coordination demands, opportunities for leader–employee interaction, and the visibility of discretionary employee behavior may differ across teams of different sizes.

Gender was dummy-coded as 0 = male and 1 = female. Age, education, and team size were represented using ordered category scores and entered as linear covariates. Exact ages, years of education, and team headcounts were not retained; therefore, these variables were not treated as genuinely continuous measures. Team size was coded 1 = 1–8 members, 2 = 9–16 members, and 3 = 17–24 members. Team size was entered as a single linear covariate using the three ordered category scores, thereby assuming a constant linear association across adjacent categories. Because exact team headcounts were unavailable, the team-size coefficient should be interpreted as the estimated association corresponding to a one-category increase in team size rather than the effect of adding one team member. To assess the robustness of the substantive findings, each principal model was estimated both with and without its respective covariates. Models 1 and 2 included leader gender, age, education, and team size at the team level, whereas Model 3 included employee gender, age, and education at the individual level in addition to the same team-level covariates.

Finally, potential common method bias was examined using Harman’s single-factor test, a single-factor measurement model comparison, and a latent common-method-factor analysis (Podsakoff et al., 2003). These analyses were conducted using the same 517 matched employee-level records as the substantive CFA. In the latent common-method-factor model, all 38 indicators used in the five-factor CFA were allowed to load simultaneously on their respective substantive factors and on an additional latent method factor. These indicators comprised six GTL items, ten environmental passion items, eleven empowerment items, seven leader-rated GOCB items, and four first-order LEI dimension indicators.

For model identification, the first loading on the latent method factor was fixed to 1, the method-factor variance was freely estimated, the method-factor mean was fixed to zero, and the covariances between the method factor and the five substantive factors were fixed to zero. The remaining method-factor loadings were freely estimated. The substantive factor loadings obtained before and after the inclusion of the method factor were compared to assess whether they changed materially.

The constructs included in this diagnostic model were not collected through one homogeneous measurement method. Employees evaluated GTL and reported empowerment in Wave 1, employees evaluated LEI and reported environmental passion in Wave 2, and team leaders evaluated employee-specific GOCB in Wave 3. The latent method factor was therefore interpreted as a broad diagnostic factor rather than as a representation of a single common source, rater, or measurement occasion. Consequently, the analysis was not treated as conclusive evidence that common method bias was absent. The method-factor variance, its standard error and exact *p*-value, and the range of standardized method-factor loadings are reported in Section 4.1.2. The comparison of substantive factor loadings before and after introducing the latent method factor is reported in Supplementary Table S2.

## 4 Data analysis and results

### 4.1 Preliminary analyses

Before testing the hypotheses, we conducted several preliminary analyses, including aggregation tests for team-level constructs, measurement model comparisons, reliability and validity assessments, common method bias diagnostics, and descriptive statistics.

#### 4.1.1 Aggregation tests

Before conducting the multilevel analyses, we examined whether employee-rated GTL and LEI could be aggregated to the team level. As shown in Table 3, both constructs showed adequate within-team agreement and between-team reliability. Specifically, GTL had an Rwg value of 0.836, an ICC (1) value of 0.419, and an ICC (2) value of 0.829. LEI had an Rwg value of 0.815, an ICC (1) value of 0.346, and an ICC (2) value of 0.780. These results indicate that employees within the same team shared sufficiently consistent perceptions of their leader’s green transformational leadership and emotional intelligence, thereby supporting the aggregation of GTL and LEI to the team level.

**Table 3:** Aggregation statistics for team-level constructs.

Construct	ICC(1)	ICC(2)	Rwg
GTL	0.419	0.829	0.836
LEI	0.346	0.780	0.815

GTL, green transformational leadership, LEI, leaders’ emotional intelligence.

#### 4.1.2 Measurement model, reliability, and convergent validity

The measurement model was estimated as a conventional single-level confirmatory factor analysis using the 517 matched employee-level records. The GTL and LEI indicators were analyzed before aggregation, whereas the leader-rated GOCB indicators were matched to the corresponding employee records using the non-identifying employee codes. Accordingly, the factor loadings reported in Table 1 represent single-level estimates rather than separate within-team or between-team estimates. As explained in Section 3.3, team clustering was not explicitly adjusted in the CFA.

The hypothesized five-factor measurement model was compared with several alternative measurement models. As shown in Table 4, among the substantive measurement models, the hypothesized five-factor model provided the best fit to the data, χ^2^ = 711.693, df = 655, χ^2^/df = 1.087, CFI = 0.996, TLI = 0.996, RMSEA = 0.013, and SRMR = 0.026. These fit indices met commonly recommended standards, with CFI and TLI exceeding 0.90 and RMSEA and SRMR falling below 0.08 (Browne and Cudeck, 1992; Hu and Bentler, 1999). The alternative four-factor, three-factor, two-factor, and one-factor models showed substantially poorer fit. These results support the empirical distinctiveness of the five focal constructs.

**Table 4:** Results of confirmatory factor analyses.

Model	χ^2^	df	χ^2^/df	RMSEA	CFI	TLI	SRMR
Five-factor model (GTL, ENV, EMP, GOCB, LEI)	711.693	655	1.087	0.013	0.996	0.996	0.026
Four-factor model (GTL + ENV, EMP, GOCB, LEI)	2623.302	659	3.981	0.076	0.855	0.845	0.119
Three-factor model (GTL + ENV + EMP, GOCB, LEI)	6298.936	662	9.515	0.128	0.583	0.558	0.147
Two-factor model (GTL + ENV + EMP + GOCB, LEI)	7331.700	664	11.042	0.139	0.507	0.478	0.147
One-factor model (GTL + ENV + EMP + GOCB+LEI)	7915.654	665	11.903	0.145	0.464	0.434	0.152
Five-factor model with latent common-method factor	640.831	617	1.039	0.009	0.998	0.998	0.023

GTL, green transformational leadership, GOCB, green organizational citizenship behavior, EMP, empowerment, ENV, environmental passion, LEI, leaders’ emotional intelligence. “+” indicates that the constructs were combined into a single factor. The latent common-method factor model refers to the hypothesized five-factor model with an additional unmeasured latent common-method factor.

Potential common method bias was assessed using preliminary diagnostic procedures and a latent common-method-factor analysis (Podsakoff et al., 2003). Harman’s single-factor test showed that the first unrotated factor explained 27.66% of the total variance. In addition, the single-factor measurement model showed substantially poorer fit than the hypothesized five-factor model. These procedures were treated only as preliminary diagnostics.

To provide a more detailed assessment, an additional latent method factor was introduced into the hypothesized five-factor model. All 38 observed indicators used in the substantive CFA—six GTL indicators, ten environmental passion indicators, eleven empowerment indicators, seven leader-rated GOCB indicators, and four first-order LEI dimension indicators—were allowed to load on both their corresponding substantive factors and an additional orthogonal method factor. As shown in Table 4, the model with the latent method factor also demonstrated good fit, χ^2^ = 640.831, df = 617, χ^2^/df = 1.039, CFI = 0.998, TLI = 0.998, RMSEA = 0.009, and SRMR = 0.023. Relative to the original five-factor model, the changes in fit indices were small: ΔCFI = 0.002, ΔTLI = 0.002, ΔRMSEA = 0.004, and ΔSRMR = 0.003.

The estimated variance of the latent method factor was 0.004 (SE = 0.016, *p* = 0.814), indicating that the overall method-factor variance was not statistically significant. The standardized method-factor loadings ranged from −0.114 to 0.328 and were generally modest in magnitude. After the latent method factor was introduced, the substantive factor loadings changed only modestly, with a mean absolute change of approximately 0.022 and a maximum absolute change of 0.058. The complete comparison of substantive factor loadings before and after introducing the latent method factor is reported in Supplementary Table S2.

Because the indicators included in this diagnostic model were collected across different waves and included both employee-reported and leader-rated measures, the additional factor should not be interpreted as representing a single homogeneous measurement source or occasion. Accordingly, the latent method-factor analysis was treated as a broad diagnostic rather than as conclusive evidence that common method bias was absent.

Reliability and convergent validity were then assessed using Cronbach’s *α*, standardized factor loadings, composite reliability (CR), and average variance extracted (AVE). As shown in Table 1, all Cronbach’s α values exceeded 0.90, all CR values exceeded 0.70, and all AVE values exceeded 0.50. The standardized substantive factor loadings were also within acceptable ranges. These results indicate satisfactory internal consistency reliability and convergent validity (Fornell and Larcker, 1981).

#### 4.1.3 Discriminant validity

Discriminant validity was assessed using the Fornell–Larcker criterion and measurement model comparisons. As shown in Table 5, the square root of the AVE for each focal construct exceeded its correlations with the other constructs at the corresponding level of analysis, supporting discriminant validity under the Fornell–Larcker criterion. In addition, the hypothesized five-factor model showed substantially better fit than the alternative models in which theoretically distinct constructs were combined. Together, these results indicate that the focal constructs were empirically distinct.

**Table 5:** Descriptive statistics, correlations, and discriminant validity.

Panel A. Individual level (*N* = 517)								
Variable	Mean	SD	1	2	3	4	5	6
Individual level								
1. Female	0.482	0.500	—					
2. Age	1.778	0.828	0.181***	—				
3. Education	1.590	0.562	0.010	0.045	—			
4. Environmental passion	3.409	1.067	0.020	−0.012	0.000	(0.837)		
5. Empowerment	3.695	0.864	−0.091*	0.031	0.008	0.286***	(0.784)	
6. Green organizational citizenship behavior	3.689	0.906	−0.014	0.030	0.005	0.389***	0.467***	(0.760)

Panel B. Team level (*N* = 77)							
Variable	Mean	SD	1	2	3	4	5
Team level							
1. Leader female	0.312	0.466	—				
2. Leader age	2.792	0.964	0.037	—			
3. Leader education	1.753	0.588	0.060	0.024	—		
4. Green transformational leadership	3.505	0.680	−0.013	0.126	0.010	(0.806)	
5. Leaders’ emotional intelligence	3.668	0.492	0.102	−0.016	−0.026	0.152	(0.722)

Panel C. Team-size distribution (*N* = 77)		
Team-size category	Number of teams	Percentage
1–8 members	5	6.49%
9–16 members	67	87.01%
17–24 members	5	6.49%

*p < 0.05, **p < 0.01, ***p < 0.001. Employee and leader gender were coded 0 = male and 1 = female. Age was coded 1 = 20–30 years, 2 = 31–40 years, 3 = 41–50 years, and 4 = over 50 years. Education was coded 1 = junior college degree or below, 2 = bachelor’s degree, and 3 = master’s degree or above. Team size was coded 1 = 1–8 members, 2 = 9–16 members, and 3 = 17–24 members. Age, education, and team size were represented using ordered category scores. Exact ages, years of education, and team headcounts were not retained; therefore, these variables should not be interpreted as genuinely continuous measures. Values in parentheses on the diagonal are the square roots of the average variance extracted for the focal constructs.

#### 4.1.4 Descriptive statistics and correlations

Descriptive statistics and zero-order correlations were calculated separately for individual-level and team-level variables to avoid conflating the two levels of analysis. As shown in Table 5, the individual-level statistics were based on 517 employee-level observations, whereas the team-level statistics were based on 77 teams after aggregating employee-rated GTL and LEI.

At the individual level, environmental passion was positively correlated with empowerment (r = 0.286, *p* < 0.001) and GOCB (r = 0.389, p < 0.001). Empowerment was also positively correlated with GOCB (r = 0.467, p < 0.001). At the team level, GTL was positively but not significantly correlated with LEI (r = 0.152, *p* = 0.187). These correlations are reported for descriptive purposes only and were not used as the basis for selecting or retaining control variables in the hypothesis-testing models.

Team size was available only in three ordered categories and was unevenly distributed across these categories, with most teams falling within the 9–16-member category. Because exact team headcounts were not retained, team size was represented using an ordered category score in the hypothesis-testing models rather than being treated as a genuinely continuous measure. The robustness of the substantive findings was further examined by comparing the controlled model with a model excluding the control variables, as reported in Supplementary Table S1.

### 4.2 Hypothesis tests

#### 4.2.1 Direct effects

The unconditional null models first partitioned the variance in environmental passion, empowerment, and GOCB into within-team and between-team components. The between-team variances were 0.695 for environmental passion, 0.271 for empowerment, and 0.331 for GOCB, whereas the corresponding within-team variances were 0.448, 0.475, and 0.475. These variance components yielded ICC values of 0.608 for environmental passion, 0.363 for empowerment, and 0.411 for GOCB. Thus, a meaningful proportion of the variance in each outcome was attributable to differences between teams, supporting the use of multilevel analysis.

As shown in Table 6, Model 1 examined environmental passion as the outcome while controlling for leader gender, leader age, leader education, and team size at the team level. GTL was positively associated with environmental passion (Estimate = 0.525, SE = 0.132, *p* < 0.001), supporting Hypothesis 4. Model 2 examined empowerment as the outcome while controlling for the same team-level covariates. GTL was positively associated with empowerment (Estimate = 0.397, SE = 0.073, *p* < 0.001), supporting Hypothesis 2.

**Table 6:** Results of multilevel path analysis.

Level	Variable/Statistic	Null Model			Model 1		Model 2		Model 3	
		ENV	EMP	GOCB	ENV	SE	EMP	SE	GOCB	SE
Intercept					0.890	0.552	1.274***	0.285	1.197**	0.454
Individual-level	Female								0.014	0.070
	Age								0.034	0.034
	Education								0.048	0.059
	ENV								0.143**	0.048
	EMP								0.308***	0.057
Team-level	Female				0.102	0.174	−0.149	0.117	0.097	0.099
	Age				0.106	0.101	−0.033	0.054	−0.032	0.048
	Education				−0.022	0.153	0.050	0.083	−0.153	0.098
	Team size				0.153	0.214	0.504***	0.136	−0.243	0.165
	GTL				0.525***	0.132	0.397***	0.073	0.267***	0.076
	LEI								0.140	0.108
	GTL × LEI								0.428*	0.177
	EMP × LEI								0.293*	0.149
Statistics	Between-team variance (τ00)	0.695	0.271	0.331	/	/	/	/	/	/
	Within-team variance (σ^2^)	0.448	0.475	0.475	0.448	0.035	0.476	0.038	/	/
	ICC	0.608	0.363	0.411	/	/	/	/	/	/
	Within-level residual variance	/	/	/	/	/	/	/	0.406	0.041
	Between-level residual variance	/	/	/	0.512	0.088	0.091	0.035	0.076	0.025

*p < 0.05, **p < 0.01, ***p < 0.001. Models 1 and 2 include leader gender, age, education, and team size as team-level covariates. Model 3 includes employee gender, age, and education at the individual level and leader gender, age, education, and team size at the team level. Gender was dummy-coded, whereas age, education, and team size were entered using ordered category scores. GTL, green transformational leadership; LEI, leaders’ emotional intelligence; ENV, environmental passion; EMP, empowerment; GOCB, green organizational citizenship behavior. Level 1 *N* = 517 employees; Level 2 *N* = 77 teams.

In Model 3, GOCB was regressed on environmental passion, empowerment, GTL, LEI, the two interaction terms, employee gender, age, and education at the individual level, and leader gender, age, education, and team size at the team level. GTL was positively associated with GOCB (Estimate = 0.267, SE = 0.076, p < 0.001), supporting Hypothesis 1. Environmental passion was positively associated with GOCB (Estimate = 0.143, SE = 0.048, *p* < 0.01), as was empowerment (Estimate = 0.308, SE = 0.057, p < 0.001). These coefficients establish the relevant component relationships, whereas the proposed indirect relationships are evaluated separately in Section 4.2.2.

Model 3 also showed a positive interaction between GTL and LEI (Estimate = 0.428, SE = 0.177, *p* < 0.05), indicating that the association between GTL and GOCB varied across levels of LEI. The interaction between empowerment and LEI was likewise positive and statistically significant (Estimate = 0.293, SE = 0.149, p < 0.05). The forms of these interactions are examined through the conditional slope analyses reported in Section 4.2.3.

To assess whether the findings were sensitive to the inclusion of the demographic and team-size covariates, the principal models were re-estimated without their respective control variables. As reported in Supplementary Table S1, GTL remained positively associated with environmental passion (Estimate = 0.593, SE = 0.099, *p* < 0.001), empowerment (Estimate = 0.567, SE = 0.063, *p* < 0.001), and GOCB (Estimate = 0.196, SE = 0.072, p < 0.01). Environmental passion (Estimate = 0.146, SE = 0.051, *p* < 0.01) and empowerment (Estimate = 0.298, SE = 0.056, *p* < 0.001) also remained positively associated with GOCB. Moreover, the GTL × LEI interaction (Estimate = 0.370, SE = 0.152, *p* < 0.05) and the empowerment × LEI interaction (Estimate = 0.288, SE = 0.145, *p* < 0.05) remained statistically significant. Accordingly, the directions and statistical conclusions of the focal direct and moderating relationships were substantively consistent across the controlled and uncontrolled specifications.

#### 4.2.2 Mediation analysis

To examine the proposed indirect relationships, we estimated the indirect effects of GTL on GOCB through empowerment and environmental passion using the product-of-coefficients approach. As shown in Table 7, the direct association between GTL and GOCB remained positive and statistically significant after empowerment and environmental passion were included in the model (Estimate = 0.267, SE = 0.076, *p* < 0.001, 95% CI [0.117, 0.416]).

**Table 7:** Mediation analysis.

Path	Effect	Estimate	SE	*p*-value	95% CI lower bound	95% CI upper bound
GTL → GOCB	Direct effect	0.267	0.076	< 0.001	0.117	0.416
GTL → EMP → GOCB	Indirect effect	0.122	0.032	< 0.001	0.059	0.186
GTL → ENV → GOCB	Indirect effect	0.075	0.031	0.015	0.014	0.136
Total indirect effect	Indirect effect	0.198	0.042	< 0.001	0.114	0.281

Entries are unstandardized estimates. Confidence intervals were calculated using the delta method. GTL, green transformational leadership; GOCB, green organizational citizenship behavior; ENV, environmental passion; EMP, empowerment; CI, confidence interval.

The indirect association between GTL and GOCB through empowerment was positive and statistically significant (Estimate = 0.122, SE = 0.032, *p* < 0.001, 95% CI [0.059, 0.186]). Because the confidence interval did not include zero, Hypothesis 3 was supported within the present model specification. The indirect association through environmental passion was also positive and statistically significant (Estimate = 0.075, SE = 0.031, *p* = 0.015, 95% CI [0.014, 0.136]), supporting Hypothesis 5.

The total indirect association through empowerment and environmental passion was statistically significant (Estimate = 0.198, SE = 0.042, p < 0.001, 95% CI [0.114, 0.281]). Taken together, these results are consistent with a partial mediation pattern: GTL was positively associated with GOCB both directly and indirectly through empowerment and environmental passion.

#### 4.2.3 Moderation analysis

**Figure 2:**
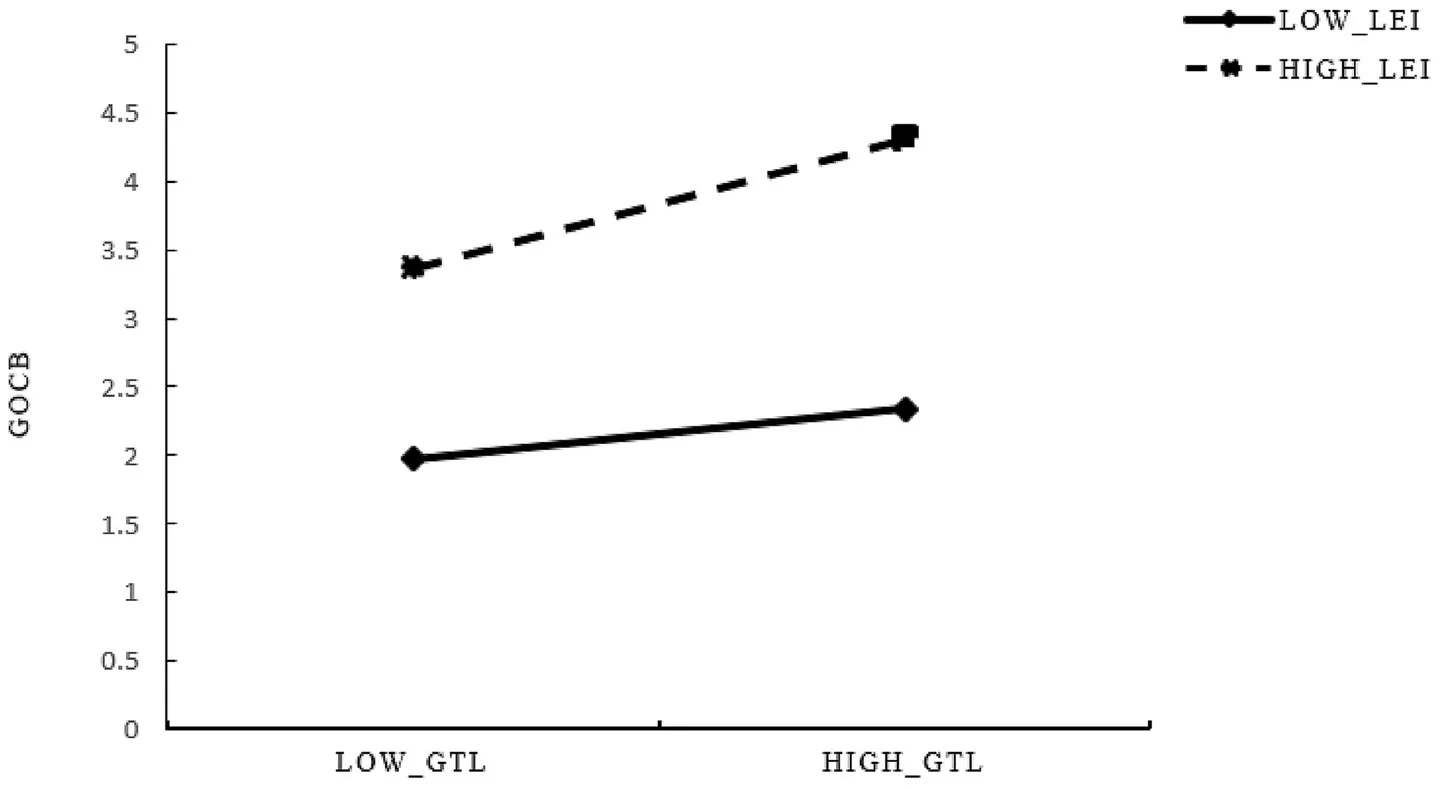
Moderating effect of LEI on the relationship between GTL and GOCB.

An interaction term between GTL and LEI was included to assess whether LEI moderates the relationship between GTL and GOCB. As reported in Table 6, the interaction between GTL and LEI was positive and statistically significant (Estimate = 0.428, SE = 0.177, p < 0.05). Simple-slope analyses further clarified this pattern (see Table 2 and Figure 2). When LEI was high, GTL was positively associated with GOCB (Estimate = 0.485, SE = 0.107, *p* < 0.001, 95% CI [0.274, 0.696]). When LEI was low, the relationship was not statistically significant (Estimate = 0.049, SE = 0.128, *p* = 0.704, 95% CI [−0.203, 0.300]). The difference between the two slopes was statistically significant (Estimate = 0.436, SE = 0.181, *p* = 0.016, 95% CI [0.081, 0.791]), supporting Hypothesis 6.

**Figure 3:**
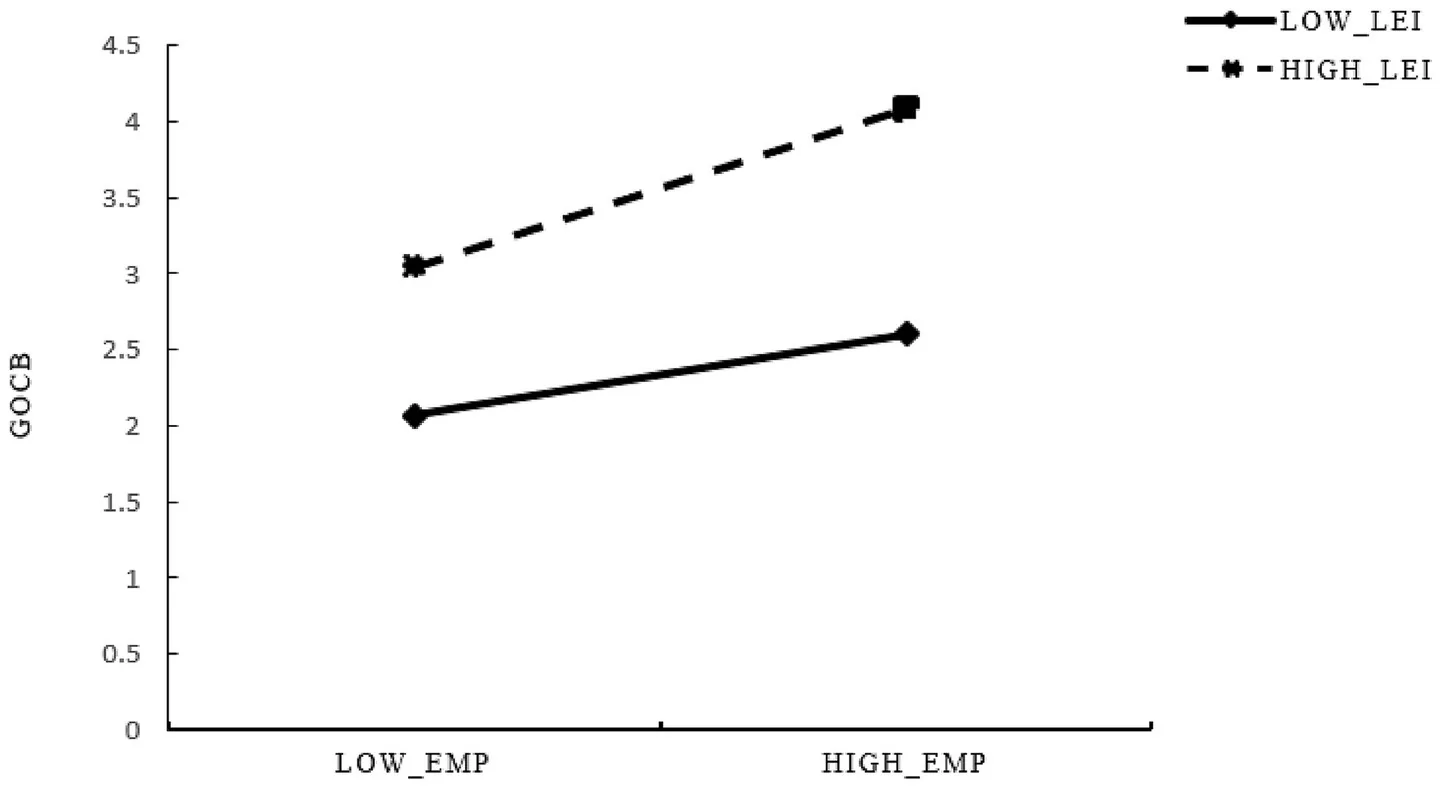
Moderating effect of LEI on the relationship between empowerment and GOCB.

A second interaction term between empowerment and LEI was incorporated to examine whether LEI moderates the relationship between empowerment and GOCB. As shown in Table 6, the interaction between empowerment and LEI was significant and positive (Estimate = 0.293, *p* = 0.049), suggesting that the relationship between empowerment and GOCB depends on the level of LEI. Simple slope analyses at high and low levels of LEI further illustrated this moderation pattern (see Table 2 and Figure 3). When LEI was high, empowerment was positively and significantly related to GOCB (Estimate = 0.457, p < 0.001, 95% CI [0.285, 0.630]). However, when LEI was low, the relationship between empowerment and GOCB was not significant (Estimate = 0.159, *p* = 0.119, 95% CI [−0.041, 0.358]). The difference between the two simple slopes was significant (Estimate = 0.299, p = 0.049, 95% CI [0.001, 0.596]). As illustrated in Figure 3, the positive slope between empowerment and GOCB was stronger when LEI was high than when LEI was low. These findings indicate that empowerment is more strongly associated with GOCB when LEI is high, thereby supporting Hypothesis 7.

## 5 Discussion

This study examined the relationships of team-level GTL with employees’ GOCB through empowerment and environmental passion, as well as the moderating role of employees’ shared perceptions of LEI. The findings showed that GTL was positively associated with GOCB and that empowerment and environmental passion accounted for significant model-based indirect associations between GTL and GOCB. Moreover, the positive relationships of GTL and empowerment with GOCB were stronger at higher levels of LEI.

Viewed through Broaden-and-Build Theory, the findings suggest that environmentally focused leadership is associated with complementary psychological resources that support discretionary green behavior. Empowerment provides employees with a broad sense of meaning, competence, self-determination, and impact in their work, whereas environmental passion provides affective energy and sustained attachment to environmental activities. LEI further shapes the socio-emotional context in which green leadership messages are received and employees exercise their work-related agency. Together, these findings offer a resource-based explanation of how team-level leadership, individual psychological resources, and the interpersonal quality of leadership are jointly associated with GOCB.

### 5.1 Theoretical implications

A more integrated account of the psychological processes associated with GTL emerges from bringing together mechanisms that prior studies have often examined separately. Existing research has highlighted green climate, moral reflectiveness, autonomous motivation, green intrinsic motivation, environmental passion, psychological empowerment, and basic psychological needs as important explanatory mechanisms (Li et al., 2020; Li et al., 2023; Liu and Yu, 2023; Zhao et al., 2024; Kaya and Atsan, 2025; Yang et al., 2025; Cao et al., 2026). Recent evidence further suggests that sustainability-oriented leadership can shape employees’ future-oriented cognition and willingness to engage in pro-environmental behavior (Nie et al., 2025). Building on this literature, the present study draws on the resource-development logic of Broaden-and-Build Theory to connect cognitive-motivational and affective mechanisms within a common explanatory framework. From this perspective, GOCB is supported by a broader configuration of psychological resources rather than by a single motivational pathway.

Second, the study advances green leadership research by distinguishing psychological resources according to their domain specificity. Empowerment captures employees’ broad experience of agency across their work roles, whereas environmental passion captures affective attachment specifically to environmental activities. Both resources accounted for significant indirect associations between GTL and GOCB when examined simultaneously. This pattern suggests that domain-general agency and domain-specific affect make complementary contributions to discretionary green action. Employees may draw on empowerment to recognize that they possess the discretion and influence required to act, while environmental passion provides the affective energy needed to sustain action beyond formal requirements. This distinction connects research on general psychological empowerment and citizenship behavior with emerging work on environmentally focused motivation and green psychological empowerment (Llorente-Alonso et al., 2024; Xu et al., 2024; Rietze et al., 2025; Yang et al., 2025). It also complements recent findings that green leadership can activate basic psychological needs and other motivational resources relevant to environmentally innovative behavior (Zhao et al., 2024).

Third, the study identifies LEI as a proximal socio-emotional boundary condition. Research on employee green behavior has predominantly emphasized structural and normative contexts such as green HRM systems, green organizational climate, and team environmental norms (Zacher et al., 2024; Dwumah et al., 2025; Zafar et al., 2025). The present findings show that the interpersonal quality of leadership also matters. GTL was more strongly associated with GOCB when leaders were collectively perceived as emotionally intelligent, suggesting that environmental messages are more influential when they are conveyed through emotionally attentive and responsive interactions. Recent multilevel evidence similarly indicates that perceived leader emotional intelligence and team emotional climate jointly shape employee motivation, flourishing, and performance (A'yuninnisa et al., 2024). Research on environmental citizenship further shows that relational conditions such as trust can strengthen the relationship between supervisory environmental support and voluntary environmental behavior (Jankelová et al., 2025). These findings position LEI as an interpersonal condition that facilitates the enactment of environmentally focused leadership.

The moderation of the empowerment–GOCB relationship further extends this contribution. Empowerment provides employees with the capacity to exercise initiative, but discretionary environmental action may involve interpersonal risk, including questioning existing routines, recommending changes, and mobilizing colleagues around activities outside formal job requirements. Higher LEI may reduce uncertainty about how such initiatives will be interpreted and increase employees’ confidence that constructive intentions will be recognized. The findings therefore suggest that the behavioral expression of empowerment depends partly on the socio-emotional environment created by the immediate leader. This connects research on psychological empowerment and constructive citizenship behavior with growing evidence that leader emotional intelligence shapes relational and affective processes within teams (Lee et al., 2023; A'yuninnisa et al., 2024; Xu et al., 2024).

Fourth, the study contributes to the multilevel literature by linking shared team-level leadership perceptions with individual-level psychological resources and leader-rated GOCB. Recent integrative research emphasizes that employee green behavior emerges from influences operating across individual, team, leadership, HRM, and organizational levels (Tang et al., 2023; Zacher et al., 2024). For example, Mo et al. (2025) showed that environmentally specific transformational leadership and green HRM jointly shape team green behavior through team-level climate processes. The present study complements this research by showing that shared perceptions of GTL and LEI are associated with individual GOCB through employees’ cognitive-motivational and affective resources. This multilevel configuration helps explain how a common team leadership context can be associated with variation in discretionary environmental behavior among individual employees.

### 5.2 Practical implications

This study provides several concrete implications for organizations seeking to promote employees’ GOCB, particularly new energy enterprises in which environmental strategies must be implemented through everyday team activities.

First, organizations should embed GTL into routine team management rather than rely on periodic sustainability campaigns. Corporate environmental objectives can be translated into a limited number of team-specific priorities and incorporated into project planning, operational meetings, work reviews, and resource-allocation decisions. Managers should regularly explain how employees’ tasks contribute to emissions reduction, resource conservation, waste prevention, or clean-energy development and should demonstrate environmentally responsible choices in their own work. Leader assessment can include observable behaviors such as communicating a clear environmental vision, inviting green suggestions, challenging environmentally inefficient routines, and recognizing employees’ voluntary contributions (Kaya and Atsan, 2025; Rietze et al., 2025). Environmental objectives should also be assigned clear responsibilities, implementation schedules, and progress indicators so that sustainability remains part of daily managerial practice rather than an abstract organizational commitment.

Second, organizations should provide employees with meaningful discretion and accessible channels for implementing environmental improvements. Employees can be authorized to modify low-risk work procedures, test resource-saving ideas, and coordinate small-scale environmental initiatives within clearly defined boundaries. Simplified approval procedures, small pilot budgets, designated proposal owners, and fixed review deadlines can reduce the administrative barriers surrounding employee-led initiatives. Green suggestion systems should display whether each proposal is under review, approved for testing, implemented, postponed, or rejected, together with the responsible manager and reasons for the decision. Participative decision-making and visible managerial follow-through can strengthen employees’ sense that their ideas influence actual work outcomes (Llorente-Alonso et al., 2024; Xu et al., 2024). Regular opportunities to raise environmental concerns can also be incorporated into team meetings and project reviews rather than being confined to occasional suggestion campaigns (Kühner et al., 2025).

Third, organizations should make the environmental value of employees’ work visible and personally meaningful. Project dashboards and post-project reviews can report concrete outcomes such as energy savings, avoided emissions, reductions in material waste, recycling improvements, and gains in resource efficiency. These outcomes should be linked to the teams and employees whose initiatives contributed to them. Managers can also use internal case presentations, environmental project rotations, and cross-functional improvement teams to expose employees to the broader environmental consequences of their work. Constructive feedback should emphasize why an employee’s contribution matters to the organization’s environmental objectives, while recognition can include opportunities to lead subsequent green projects, share successful practices, or participate in strategic sustainability initiatives (Iqbal et al., 2023). Recognition systems should preserve the voluntary character of GOCB rather than convert discretionary environmental contributions into compulsory performance quotas (Abourokbah et al., 2024; Cao et al., 2026).

Finally, organizations should manage the environmental and socio-emotional dimensions of leadership jointly. Employee upward-feedback surveys can assess whether managers not only communicate strong environmental priorities but also listen attentively, acknowledge concerns, regulate their reactions, and respond constructively to employee initiative. This approach can identify managers whose environmental direction is strong but whose interpersonal style discourages employees from proposing changes or acting beyond formal duties. Leader evaluation should therefore include response standards such as timely acknowledgment of employee proposals, clear explanations when ideas cannot be implemented, respectful handling of disagreement, and non-punitive responses to responsible experiments that fail. Perceived leader emotional intelligence is particularly relevant to employees’ experience of team interaction and should be assessed through employee feedback rather than leader self-evaluation alone (Hu et al., 2023; A'yuninnisa et al., 2024). Coaching and developmental assessment can then focus on realistic environmental-change scenarios involving workload concerns, resistance to new procedures, unsuccessful green initiatives, and conflict over operational priorities (Ertiö et al., 2024).

Overall, organizations should combine clear environmental direction, meaningful employee discretion, visible environmental outcomes, and constructive leader responses. Integrating these practices into ordinary team-management systems provides a more sustainable basis for GOCB than relying solely on formal environmental policies or generic leadership training.

### 5.3 Limitations and future research directions

Several limitations suggest directions for future research. First, the sample was drawn from new energy enterprises in three regions of China. Although this context is appropriate because environmental goals are particularly salient in such firms, it may limit the generalizability of the findings. Future studies should replicate the model in other environmentally sensitive industries and institutional settings. Cross-cultural research is also needed because responses to GTL, empowerment, environmental passion, and LEI may vary across cultural contexts (Zacher et al., 2024).

Second, the three-wave time-lagged design reduces concerns associated with cross-sectional data but does not establish causality. The observation period was also relatively short. Future research could employ longer-term longitudinal, cross-lagged, or experience-sampling designs to examine how the focal relationships develop over time and whether reciprocal effects occur. For example, employees’ sustained engagement in GOCB may subsequently encourage leaders to display stronger GTL.

Third, several focal variables remained perceptual measures. GTL, empowerment, environmental passion, and LEI were assessed by employees, whereas GOCB was rated by team leaders. In particular, LEI represents employees’ shared perceptions of their leaders’ emotional intelligence rather than an objective ability-based assessment. In addition, although the measurement-model comparison and latent common-method factor analysis did not indicate that substantial common method variance explained the findings, the survey did not include a theoretically unrelated marker variable. Future studies should combine ability-based EI measures, multiple raters, marker-variable designs, and objective environmental indicators, such as energy-use, waste-reduction, or environmental-audit records.

Finally, this study focused on empowerment and environmental passion as psychological mechanisms and LEI as the primary boundary condition. Future research could compare these mechanisms with green intrinsic motivation, green self-efficacy, or green work meaningfulness (Kaya and Atsan, 2025; Rietze et al., 2025). It could also directly compare LEI with alternative contextual moderators emphasized in the broader literature, including green organizational climate, green HRM practices, team environmental norms, and environmental strategy implementation (Dwumah et al., 2025; Zacher et al., 2024). Such comparisons would help clarify when socio-emotional leadership conditions provide explanatory value beyond more formal organizational and team-level environmental contexts.

## Data Availability

The raw data supporting the conclusions of this article will be made available by the authors, without undue reservation.

## References

[ref1] Abdullahi A. Z. Anarfo E. B. Anyigba H. (2020). The impact of leadership style on organizational citizenship behavior: does leaders’ emotional intelligence play a moderating role? J. Manag. Dev. 39, 963–987. doi: 10.1108/JMD-01-2020-0012

[ref2] Abourokbah S. Bajaba S. Yaqub M. Z. (2024). Leading the green wave: how and when green transformational leadership cultivates employee green creativity. Acta Psychol. 250:104503. doi: 10.1016/j.actpsy.2024.104503, 39357415

[ref3] Alwheshi A. Alzubi A. Iyiola K. (2024). Linking responsible leadership to organizational citizenship behavior for the environment: examining the role of employees harmonious environmental passion and environmental transformational leadership. SAGE Open 14:21582440241271177. doi: 10.1177/21582440241271177

[ref4] Ayoko O. B. Tan P. P. Li Y. (2023). Leader–follower interpersonal behaviors, emotional regulation and LMX quality. J. Manag. Organ. 29, 553–570. doi: 10.1017/jmo.2022.26

[ref5] A'yuninnisa R. N. Carminati L. Wilderom C. P. M. (2024). Promoting employee flourishing and performance: the roles of perceived leader emotional intelligence, positive team emotional climate, and employee emotional intelligence. Front. Organ. Psychol. 2:1283067. doi: 10.3389/forgp.2024.1283067

[ref6] Becker T. E. (2005). Potential problems in the statistical control of variables in organizational research: a qualitative analysis with recommendations. Organ. Res. Methods 8, 274–289. doi: 10.1177/1094428105278021

[ref7] Bernerth J. B. Aguinis H. (2016). A critical review and best-practice recommendations for control variable usage. Pers. Psychol. 69, 229–283. doi: 10.1111/peps.12103

[ref8] Boiral O. (2009). Greening the corporation through organizational citizenship behaviors. J. Bus. Ethics 87, 221–236. doi: 10.1007/s10551-008-9881-2

[ref9] Boiral O. Paillé P. (2012). Organizational citizenship behaviour for the environment: measurement and validation. J. Bus. Ethics 109, 431–445. doi: 10.1007/s10551-011-1138-9

[ref10] Browne M. W. Cudeck R. (1992). Alternative ways of assessing model fit. Sociol. Methods Res. 21, 230–258. doi: 10.1177/0049124192021002005

[ref11] Cao J. Sun H. Cao X. Ma H. (2026). Green transformational leadership and employees’ green behavior: a cross-level moderated mediation study. Front. Psychol. 17:1720404. doi: 10.3389/fpsyg.2026.1720404, 41743437 PMC12929164

[ref12] Chen Y.-S. Chang C.-H. (2013). The determinants of green product development performance: green dynamic capabilities, green transformational leadership, and green creativity. J. Bus. Ethics 116, 107–119. doi: 10.1007/s10551-012-1452-x

[ref13] Cronin M. A. Stouten J. Van Knippenberg D. (2021). The theory crisis in management research: solving the right problem. Acad. Manag. Rev. 46, 667–683. doi: 10.5465/amr.2019.0294, 42475469

[ref14] Dwumah K. Sappor P. Sarpong F. A. (2025). Navigating the green path: strategies for enhancing organizational citizenship behavior for the environment through green HRM practices. Bus. Strateg. Environ. 34, 3045–3061. doi: 10.1002/bse.4149

[ref15] Enders C. K. Tofighi D. (2007). Centering predictor variables in cross-sectional multilevel models: a new look at an old issue. Psychol. Methods 12, 121–138. doi: 10.1037/1082-989X.12.2.121, 17563168

[ref16] Ertiö T. Eriksson T. Rowan W. McCarthy S. (2024). The role of digital leaders’ emotional intelligence in mitigating employee technostress. Bus. Horiz. 67, 399–409. doi: 10.1016/j.bushor.2024.03.004

[ref17] Fornell C. Larcker D. F. (1981). Evaluating structural equation models with unobservable variables and measurement error. J. Mark. Res. 18, 39–50. doi: 10.1177/002224378101800104

[ref18] Fredrickson B. L. (2001). The role of positive emotions in positive psychology: the broaden-and-build theory of positive emotions. Am. Psychol. 56, 218–226. doi: 10.1037/0003-066X.56.3.218, 11315248 PMC3122271

[ref19] Fredrickson B. L. Branigan C. (2005). Positive emotions broaden the scope of attention and thought-action repertoires. Cogn. Emot. 19, 313–332. doi: 10.1080/02699930441000238, 21852891 PMC3156609

[ref20] Fredrickson B. L. Cohn M. A. Coffey K. A. Pek J. Finkel S. M. (2008). Open hearts build lives: positive emotions, induced through loving-kindness meditation, build consequential personal resources. J. Pers. Soc. Psychol. 95, 1045–1062. doi: 10.1037/a0013262, 18954193 PMC3156028

[ref21] Fu L. Tang J. Zhou H. Zeng G. (2025). Inclusive climate or innovative climate? The mechanism of green transformational leadership motivating green mindfulness. J. Environ. Manag. 378:124750. doi: 10.1016/j.jenvman.2025.124750, 40037248

[ref22] Hancer M. George R. T. (2003). Psychological empowerment of non-supervisory employees working in full-service restaurants. Int. J. Hosp. Manag. 22, 3–16. doi: 10.1016/S0278-4319(02)00032-4

[ref23] Hu W.-H. (2025). Exploring the impact of psychological empowerment on customer orientation through psychological safety: the role of professional commitment as a moderator. BMC Psychol. 13:1028. doi: 10.1186/s40359-025-03345-0, 41013650 PMC12465526

[ref24] Hu L. Bentler P. M. (1999). Cutoff criteria for fit indexes in covariance structure analysis: conventional criteria versus new alternatives. Struct. Equ. Model. Multidiscip. J. 6, 1–55. doi: 10.1080/10705519909540118

[ref25] Hu X. Li R. Y. M. Kumari K. Ben Belgacem S. Fu Q. Khan M. A. . (2023). Relationship between green leaders’ emotional intelligence and employees’ green behavior: a PLS-SEM approach. Behav. Sci. 13:25. doi: 10.3390/bs13010025, 36661597 PMC9854950

[ref26] Iftikhar Y. Tufail M. S. Ferasso M. Danish R. Q. (2024). Servant leadership and citizenship behavior in the Pakistani tourism and hospitality industry: the role of harmonious environmental passion and a green work climate. J. Environ. Manag. 369:122276. doi: 10.1016/j.jenvman.2024.122276, 39197345

[ref27] Ilies R. Bono J. E. Bakker A. B. (2024). Crafting well-being: employees can enhance their own well-being by savoring, reflecting upon, and capitalizing on positive work experiences. Annu. Rev. Organ. Psychol. Organ. Behav. 11, 63–91. doi: 10.1146/annurev-orgpsych-110721-045931

[ref28] Iqbal R. Shahzad K. Donia M. B. L. (2023). Environmentally specific transformational leadership and employee green attitude and behavior: an affective events theory perspective. J. Environ. Psychol. 92:102181. doi: 10.1016/j.jenvp.2023.102181

[ref29] James L. R. Demaree R. G. Wolf G. (1984). Estimating within-group interrater reliability with and without response bias. J. Appl. Psychol. 69, 85–98. doi: 10.1037/0021-9010.69.1.85

[ref30] Jankelová N. Némethová I. Dabić M. Kallmuenzer A. (2025). Enhancing organizational citizenship behavior towards the environment. Rev. Manag. Sci. 19, 899–930. doi: 10.1007/s11846-024-00781-x, 30311153

[ref31] Kaya N. Atsan N. (2025). How green transformational leadership influences employee green behavior: the mediating role of green intrinsic motivation. J. Environ. Manag. 396:128020. doi: 10.1016/j.jenvman.2025.128020, 41274273

[ref32] Khan A. N. Khan N. A. (2022). The nexuses between transformational leadership and employee green organisational citizenship behaviour: role of environmental attitude and green dedication. Bus. Strateg. Environ. 31, 921–933. doi: 10.1002/bse.2926

[ref33] Klein K. J. Kozlowski S. W. J. (2000). From micro to meso: critical steps in conceptualizing and conducting multilevel research. Organ. Res. Methods 3, 211–236. doi: 10.1177/109442810033001

[ref34] Kühner C. Stein M. Zacher H. Weiss M. (2025). Employee environmental voice shapes environmental attitudes and green organizational climate (but not vice versa): a 1-year, 5-wave longitudinal study. J. Environ. Psychol. 107:102756. doi: 10.1016/j.jenvp.2025.102756

[ref35] LeBreton J. M. Senter J. L. (2008). Answers to 20 questions about interrater reliability and interrater agreement. Organ. Res. Methods 11, 815–852. doi: 10.1177/1094428106296642

[ref36] Lee C.-C. Yeh W.-C. Yu Z. Lin X.-C. (2023). The relationships between leader emotional intelligence, transformational leadership, and transactional leadership and job performance: a mediator model of trust. Heliyon 9:e18007. doi: 10.1016/j.heliyon.2023.e18007, 37534000 PMC10391948

[ref37] Li Z. Liang L. Zhang X. Li J. (2023). Impact of environmentally specific transformational leadership on organizational citizenship behavior for the environment: the role of moral reflectiveness and leader group prototypicality. J. Environ. Plan. Manag. 66, 1413–1430. doi: 10.1080/09640568.2022.2027748

[ref38] Li Z. Xue J. Li R. Chen H. Wang T. (2020). Environmentally specific transformational leadership and employee’s pro-environmental behavior: the mediating roles of environmental passion and autonomous motivation. Front. Psychol. 11:1408. doi: 10.3389/fpsyg.2020.01408, 32670165 PMC7330121

[ref39] Liu X. Yu X. (2023). Green transformational leadership and employee organizational citizenship behavior for the environment in the manufacturing industry: a social information processing perspective. Front. Psychol. 13:1097655. doi: 10.3389/fpsyg.2022.1097655, 36743625 PMC9891139

[ref40] Llorente-Alonso M. García-Ael C. Topa G. (2024). A meta-analysis of psychological empowerment: antecedents, organizational outcomes, and moderating variables. Curr. Psychol. 43, 1759–1784. doi: 10.1007/s12144-023-04369-8

[ref41] Mayer J. D. Caruso D. R. Salovey P. (2016). The ability model of emotional intelligence: principles and updates. Emot. Rev. 8, 290–300. doi: 10.1177/1754073916639667

[ref42] Miao C. Humphrey R. H. Qian S. (2018). A cross-cultural meta-analysis of how leader emotional intelligence influences subordinate task performance and organizational citizenship behavior. J. World Bus. 53, 463–474. doi: 10.1016/j.jwb.2018.01.003

[ref43] Mo Z. Liu M. T. Lai I. K. W. (2025). The dynamic joint roles of green human resource management and environmentally specific transformational leadership on team green behavior. Tour. Manag. 107:105046. doi: 10.1016/j.tourman.2024.105046

[ref44] Nie T. Lan M. Mo D. (2025). Follow and go beyond: the impact of sustainable leadership on employees’ pro-environmental behaviors. J. Environ. Psychol. 106:102745. doi: 10.1016/j.jenvp.2025.102745

[ref45] Oktaysoy O. Topcuoglu E. Selvi M. S. Uygungil-Erdogan S. Şahin Y. Tatar V. . (2025). Psychological green climate as a mediator between green transformational leadership, innovation, and environmental awareness. Front. Psychol. 16:1701658. doi: 10.3389/fpsyg.2025.1701658, 41356049 PMC12676600

[ref46] Podsakoff P. M. MacKenzie S. B. Lee J.-Y. Podsakoff N. P. (2003). Common method biases in behavioral research: a critical review of the literature and recommended remedies. J. Appl. Psychol. 88, 879–903. doi: 10.1037/0021-9010.88.5.879, 14516251

[ref47] Preacher K. J. Zyphur M. J. Zhang Z. (2010). A general multilevel SEM framework for assessing multilevel mediation. Psychol. Methods 15, 209–233. doi: 10.1037/a0020141, 20822249

[ref48] Rietze S. Kühner C. Zacher H. (2025). Green transformational leadership and green voice behavior: the motivational role of green psychological empowerment. Corp. Soc. Responsib. Environ. Manag. 32, 7727–7743. doi: 10.1002/csr.70104, 41531421

[ref49] Robertson J. L. (2018). The nature, measurement and nomological network of environmentally specific transformational leadership. J. Bus. Ethics 151, 961–975. doi: 10.1007/s10551-017-3569-4

[ref50] Robertson J. L. Barling J. (2013). Greening organizations through leaders’ influence on employees’ pro-environmental behaviors. J. Organ. Behav. 34, 176–194. doi: 10.1002/job.1820

[ref51] Schermuly C. C. Creon L. Gerlach P. Graßmann C. Koch J. (2022). Leadership styles and psychological empowerment: a meta-analysis. J. Leadersh. Organ. Stud. 29, 73–95. doi: 10.1177/15480518211067751

[ref52] Seibert S. E. Wang G. Courtright S. H. (2011). Antecedents and consequences of psychological and team empowerment in organizations: a meta-analytic review. J. Appl. Psychol. 96, 981–1003. doi: 10.1037/a0022676, 21443317

[ref53] Spreitzer G. M. (1995). Psychological empowerment in the workplace: dimensions, measurement, and validation. Acad. Manag. J. 38, 1442–1465. doi: 10.2307/256865

[ref54] Tang G. Ren S. Wang M. Li Y. Zhang S. (2023). Employee green behaviour: a review and recommendations for future research. Int. J. Manag. Rev. 25, 297–317. doi: 10.1111/ijmr.12328

[ref55] Vallerand R. J. Blanchard C. Mageau G. A. Koestner R. Ratelle C. Léonard M. . (2003). Les passions de l’âme: On obsessive and harmonious passion. J. Pers. Soc. Psychol. 85, 756–767. doi: 10.1037/0022-3514.85.4.756, 14561128

[ref56] Wagner D. G. Berger J. (1985). Do sociological theories grow? Am. J. Sociol. 90, 697–728. doi: 10.1086/228142

[ref57] Wong C.-S. Law K. S. (2002). The effects of leader and follower emotional intelligence on performance and attitude. Leadersh. Q. 13, 243–274. doi: 10.1016/S1048-9843(02)00099-1

[ref58] Xing S. Yu X. Yu J. Chen J. (2026). How leadership emotional intelligence promotes team innovation: parallel mediating roles of psychological safety and knowledge sharing. Front. Psychol. 17:1806655. doi: 10.3389/fpsyg.2026.1806655, 42183528 PMC13194025

[ref59] Xu Q. Liu S. Huang H. (2024). Psychological empowerment and challenge-oriented organizational citizenship behavior: a dual process model. Front. Psychol. 15:1432260. doi: 10.3389/fpsyg.2024.1432260, 39726614 PMC11670482

[ref60] Yang N. Gao J. Chang P.-C. (2025). Can environmentally-specific transformational leadership foster employees’ green voice behavior? A moderated mediation model of psychological empowerment, ecological reflexivity, and value congruence. Behav. Sci. 15:945. doi: 10.3390/bs15070945, 40723729 PMC12292308

[ref61] Zacher H. Kühner C. Katz I. M. Rudolph C. W. (2024). Leadership and environmental sustainability: an integrative conceptual model of multilevel antecedents and consequences of leader green behavior. Group Organ. Manag. 49, 365–394. doi: 10.1177/10596011241229891

[ref62] Zafar H. Tian F. Ho J. A. Roh T. Latif B. (2025). Understanding voluntary pro-environmental behavior among colleagues: roles of green crafting, psychological empowerment, and green organizational climate. Bus. Strateg. Environ. 34, 468–482. doi: 10.1002/bse.4001

[ref63] Zaid W. M. A. Yaqub M. Z. (2024). The prolificacy of green transformational leadership in shaping employee green behavior during times of crises in small and medium enterprises: a moderated mediation model. Front. Psychol. 15:1258990. doi: 10.3389/fpsyg.2024.1258990, 38464624 PMC10920347

[ref64] Zhao F. Zhu H. Chen Y. (2024). Due responsibility and true responsibility: a moderated-mediation model linking green-harmonious human resource practice to employee organizational citizenship behavior. Asia Pac. J. Hum. Resour. 62:e12399. doi: 10.1111/1744-7941.12399

