# Supplementary material for: Building psychological resources for green organizational citizenship behavior: a broaden-and-build perspective on empowerment, environmental passion, and leaders’ emotional intelligence
Source: Front Psychol. 2026 Aug 18;17:1868718. doi: 10.3389/fpsyg.2026.1868718 (PMC13529448; doi:10.3389/fpsyg.2026.1868718)
Supplement: Supplementary file 1 [file Table_1.docx]

**Supplementary Table S1.**

Sensitivity Analysis Comparing Models With and Without Control Variables

| **Relationship** | **With controls, Estimate (SE)** | **Without controls, Estimate (SE)** |
| --- | --- | --- |
| GTL → ENV | 0.525*** (0.132) | 0.593*** (0.099) |
| GTL → EMP | 0.397*** (0.073) | 0.567*** (0.063) |
| GTL → GOCB | 0.267*** (0.076) | 0.196** (0.072) |
| ENV → GOCB | 0.143** (0.048) | 0.146** (0.051) |
| EMP → GOCB | 0.308*** (0.057) | 0.298*** (0.056) |
| LEI → GOCB | 0.140 (0.108) | 0.139 (0.111) |
| GTL × LEI → GOCB | 0.428* (0.177) | 0.370* (0.152) |
| EMP × LEI → GOCB | 0.293* (0.149) | 0.288* (0.145) |

Note: Entries are unstandardized coefficient estimates, with standard errors reported in parentheses. The model with controls included employee gender, age, and education at the individual level and leader gender, age, education, and team size at the team level. The model without controls omitted these control variables but retained the focal predictors, the main effect of LEI, and the interaction terms. Both models were estimated using maximum likelihood based on 517 employees nested within 77 teams. The substantive conclusions remained unchanged across the two specifications. GTL = green transformational leadership; ENV = environmental passion; EMP = empowerment; GOCB = green organizational citizenship behavior; LEI = leaders’ emotional intelligence. p < 0.05, p < 0.01, p < 0.001.
