# Supplementary material for: Building psychological resources for green organizational citizenship behavior: a broaden-and-build perspective on empowerment, environmental passion, and leaders’ emotional intelligence
Source: Front Psychol. 2026 Aug 18;17:1868718. doi: 10.3389/fpsyg.2026.1868718 (PMC13529448; doi:10.3389/fpsyg.2026.1868718)
Supplement: Supplementary file 2 [file Table_2.docx]

**Supplementary Table S2.**

Comparison of Substantive Factor Loadings Before and After Introducing the Latent Common-Method Factor

|  | Without method factor | | | | With method factor | | | |
| --- | --- | --- | --- | --- | --- | --- | --- | --- |
| Indicator | Estimate | S.E. | Est./S.E. | p-Value | Estimate | S.E. | Est./S.E. | p-Value |
| GTL1 | 0.824 | 0.017 | 49.926 | < 0.001 | 0.825 | 0.018 | 46.563 | < 0.001 |
| GTL2 | 0.794 | 0.019 | 42.767 | < 0.001 | 0.793 | 0.020 | 39.321 | < 0.001 |
| GTL3 | 0.826 | 0.016 | 50.310 | < 0.001 | 0.827 | 0.021 | 38.467 | < 0.001 |
| GTL4 | 0.791 | 0.019 | 42.186 | < 0.001 | 0.789 | 0.019 | 41.376 | < 0.001 |
| GTL5 | 0.800 | 0.018 | 44.206 | < 0.001 | 0.801 | 0.018 | 43.586 | < 0.001 |
| GTL6 | 0.799 | 0.018 | 44.015 | < 0.001 | 0.798 | 0.019 | 41.695 | < 0.001 |
| ENV1 | 0.826 | 0.015 | 55.147 | < 0.001 | 0.824 | 0.021 | 39.488 | < 0.001 |
| ENV2 | 0.850 | 0.013 | 64.165 | < 0.001 | 0.842 | 0.019 | 43.500 | < 0.001 |
| ENV3 | 0.853 | 0.013 | 65.380 | < 0.001 | 0.844 | 0.020 | 41.320 | < 0.001 |
| ENV4 | 0.797 | 0.017 | 46.671 | < 0.001 | 0.772 | 0.031 | 24.987 | < 0.001 |
| ENV5 | 0.857 | 0.013 | 67.057 | < 0.001 | 0.840 | 0.022 | 38.508 | < 0.001 |
| ENV6 | 0.846 | 0.014 | 62.303 | < 0.001 | 0.830 | 0.023 | 35.385 | < 0.001 |
| ENV7 | 0.867 | 0.012 | 72.017 | < 0.001 | 0.845 | 0.023 | 36.542 | < 0.001 |
| ENV8 | 0.807 | 0.016 | 49.434 | < 0.001 | 0.789 | 0.026 | 29.882 | < 0.001 |
| ENV9 | 0.826 | 0.015 | 54.929 | < 0.001 | 0.791 | 0.032 | 24.706 | < 0.001 |
| ENV10 | 0.834 | 0.014 | 57.969 | < 0.001 | 0.820 | 0.023 | 35.893 | < 0.001 |
| EMP1 | 0.778 | 0.019 | 41.596 | < 0.001 | 0.801 | 0.026 | 30.729 | < 0.001 |
| EMP2 | 0.803 | 0.017 | 47.297 | < 0.001 | 0.764 | 0.036 | 21.074 | < 0.001 |
| EMP3 | 0.804 | 0.017 | 47.413 | < 0.001 | 0.784 | 0.029 | 26.613 | < 0.001 |
| EMP4 | 0.761 | 0.020 | 38.394 | < 0.001 | 0.731 | 0.035 | 20.896 | < 0.001 |
| EMP5 | 0.779 | 0.019 | 41.703 | < 0.001 | 0.772 | 0.025 | 31.400 | < 0.001 |
| EMP6 | 0.778 | 0.019 | 41.533 | < 0.001 | 0.745 | 0.036 | 20.515 | < 0.001 |
| EMP7 | 0.803 | 0.017 | 47.256 | < 0.001 | 0.759 | 0.038 | 20.093 | < 0.001 |
| EMP8 | 0.762 | 0.020 | 38.448 | < 0.001 | 0.704 | 0.045 | 15.701 | < 0.001 |
| EMP9 | 0.773 | 0.019 | 40.656 | < 0.001 | 0.764 | 0.027 | 28.604 | < 0.001 |
| EMP10 | 0.786 | 0.018 | 43.386 | < 0.001 | 0.759 | 0.033 | 23.145 | < 0.001 |
| EMP11 | 0.800 | 0.017 | 46.408 | < 0.001 | 0.774 | 0.032 | 24.008 | < 0.001 |
| GOCB1 | 0.771 | 0.020 | 37.828 | < 0.001 | 0.797 | 0.026 | 30.156 | < 0.001 |
| GOCB2 | 0.766 | 0.021 | 36.941 | < 0.001 | 0.724 | 0.045 | 16.121 | < 0.001 |
| GOCB3 | 0.776 | 0.020 | 38.582 | < 0.001 | 0.780 | 0.027 | 28.476 | < 0.001 |
| GOCB4 | 0.732 | 0.023 | 32.019 | < 0.001 | 0.694 | 0.047 | 14.832 | < 0.001 |
| GOCB5 | 0.756 | 0.021 | 35.364 | < 0.001 | 0.751 | 0.030 | 25.370 | < 0.001 |
| GOCB6 | 0.768 | 0.021 | 37.218 | < 0.001 | 0.729 | 0.049 | 14.981 | < 0.001 |
| GOCB7 | 0.745 | 0.022 | 33.696 | < 0.001 | 0.703 | 0.052 | 13.589 | < 0.001 |
| LEI1 | 0.739 | 0.027 | 27.162 | < 0.001 | 0.696 | 0.042 | 16.544 | < 0.001 |
| LEI2 | 0.728 | 0.028 | 26.239 | < 0.001 | 0.693 | 0.049 | 14.083 | < 0.001 |
| LEI3 | 0.726 | 0.028 | 26.176 | < 0.001 | 0.709 | 0.041 | 17.278 | < 0.001 |
| LEI4 | 0.692 | 0.029 | 23.533 | < 0.001 | 0.640 | 0.055 | 11.582 | < 0.001 |

Note: “Without method factor” reports the standardized substantive factor loadings from the hypothesized five-factor model. “With method factor” reports the corresponding substantive factor loadings after an additional latent common-method factor was introduced. The substantive factor loadings changed only modestly after the method factor was added; the mean absolute change was approximately 0.022, and the maximum absolute change was 0.058. All reported loading estimates were statistically significant at *p* < 0.001.
